# Open-source, high-throughput targeted *in situ* transcriptomics for developmental and tissue biology

**DOI:** 10.1242/dev.202448

**Published:** 2024-08-29

**Authors:** Hower Lee, Christoffer Mattsson Langseth, Sergio Marco Salas, Sanem Sariyar, Andreas Metousis, Eneritz Rueda-Alaña, Christina Bekiari, Emma Lundberg, Fernando García-Moreno, Marco Grillo, Mats Nilsson

**Affiliations:** ^1^ Science for Life Laboratory, Department of Biochemistry and Biophysics, Stockholm University, 171 65 Solna, Sweden; ^2^ Science for Life Laboratory, Department of Protein Science, KTH - Royal Institute of Technology, 17165 Stockholm, Sweden; ^3^ Achucarro Basque Center for Neuroscience, Scientific Park of the University of the Basque Country (UPV/EHU), 48940 Leioa, Spain; ^4^ Department of Neuroscience, Faculty of Medicine and Odontology, UPV/EHU, Barrio Sarriena s/n, 48940 Leioa, Bizkaia, Spain; ^5^ IKERBASQUE Foundation, María Díaz de Haro 3, 6th Floor, 48013 Bilbao Spain; ^6^ Department of Bioengineering, Stanford University, Stanford, CA 94305, USA; ^7^ Department of Pathology, Stanford University, Stanford, CA 94305, USA

**Keywords:** Spatial transcriptomics, *In situ* hybridization, Multiplex imaging, Multi-omics, Open source, Padlock probes

## Abstract

Multiplexed spatial profiling of mRNAs has recently gained traction as a tool to explore the cellular diversity and the architecture of tissues. We propose a sensitive, open-source, simple and flexible method for the generation of *in situ* expression maps of hundreds of genes. We use direct ligation of padlock probes on mRNAs, coupled with rolling circle amplification and hybridization-based *in situ* combinatorial barcoding, to achieve high detection efficiency, high-throughput and large multiplexing. We validate the method across a number of species and show its use in combination with orthogonal methods such as antibody staining, highlighting its potential value for developmental and tissue biology studies. Finally, we provide an end-to-end computational workflow that covers the steps of probe design, image processing, data extraction, cell segmentation, clustering and annotation of cell types. By enabling easier access to high-throughput spatially resolved transcriptomics, we hope to encourage a diversity of applications and the exploration of a wide range of biological questions.

## Teaser

**Summary:** We describe an improved open-source, flexible and customizable method and complete computational workflow for high-throughput multiplexed *in situ* transcriptomics, readily applicable to a wide range of tissue types and model organisms.

## INTRODUCTION

Methods for spatial profiling of mRNAs have emerged as tools to explore and visualize cellular diversity in its spatial context (Borm et al., 2023; Chen et al., 2015; Codeluppi et al., 2018; Femino et al., 1998; Gyllborg et al., 2020; Lee et al., 2022). Some of these methods are untargeted and based on next-generation sequencing; hence, they can be easily adapted to new model organisms. Other methods are imaging based and generally targeted, requiring a pre-selection of probes to capture the expression of specific transcripts. Methods of both categories often require specialized or proprietary equipment, and their commercial implementations are usually bundled within expensive machines and kits, limiting the range of potential uses and applications, and wider adoption by a more diversified research community.

Targeted *in situ* sequencing (ISS) is a method for multiplexed mRNA detection (Gyllborg et al., 2020; Ke et al., 2013; Lee et al., 2022) that relies on the ligation of barcoded padlock probes on *in situ*-synthesized cDNAs, followed by rolling circle amplification, to generate gene-specific amplicons *in situ*. These amplicons are large (approximately 1 μm) and contain concatemers of hundreds of copies of the original probe (Lizardi et al., 1998), producing bright signals that can be imaged at low magnification when interrogated by iterative cycles of hybridization and stripping of fluorescent probes. Using a combinatorial detection scheme, the number of detectable genes scales by the rule *x^N^*, where *x* is the number of available fluorophores and *N* is the number of imaging cycles.

Compared with other *in situ* methods, ISS has relatively low detection efficiency. On the one hand, this is a desirable property: because only a small subset of the total mRNA molecules is captured and amplified, they can be easily visualized using widefield fluorescence microscopes at low magnification without overcrowding the image with signals, enabling fast imaging of large tissue sections (high throughput). As an example, a square tissue area of side 7.5 mm can be imaged (20×) in five channels in about 25 min per cycle. On the other hand, ISS detection of low-expressed transcripts can sometimes be challenging. Finally, most imaging-based spatial omics methods produce large (terabyte-sized) and complex image datasets that are often cumbersome to analyze.

To address these limitations, we first introduced a new detection chemistry with increased capture efficiency and validated it on a number of use cases. Second, we compiled a complete analysis pipeline, covering the steps of probe design, image analysis, data mining, decoding, cell segmentation and clustering. We also produced a complete manual to guide new users through an entire ISS experiment, and we detail how to adapt our core analysis to different microscopes or input data format. We hope that this work will encourage a larger and diverse research community to experiment with spatially resolved omics techniques for addressing a wider range of scientific questions.

## RESULTS AND DISCUSSION

### RNA-ISS recapitulates known mRNA expression patterns with high sensitivity

**Fig. 1.**
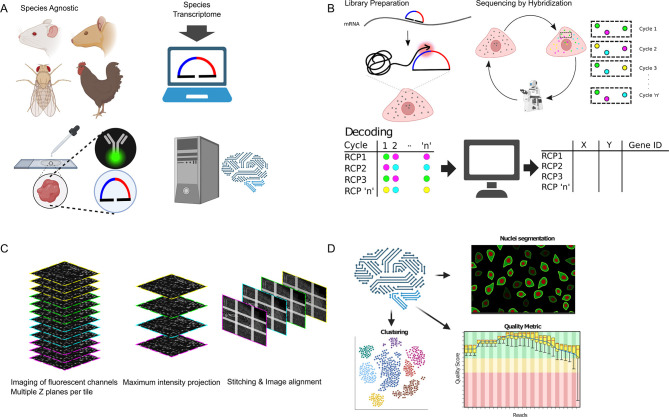
**Open-source, high-throughput targeted *in situ* transcriptomics.** (A) The open-source RNA *in situ* sequencing (ISS) assay is species agnostic and is compatible with immunohistochemistry and other fluorescence labeling, after transcript detection. The workflow includes probe design and image processing pipelines. (B) Overview of RNA-ISS. First, gene-specific chimeric padlock probes hybridize to their complementary mRNA target sequence, before they are ligated and amplified by rolling circle amplification. Next, the *in situ*-generated rolling circle products (RCPs) are combinatorially labeled, imaged across multiple cycles and computationally decoded to identify the corresponding genes. (C) Overview of the pre-processing pipeline. Raw images are transformed into a format suitable for decoding. First, the images are maximum-intensity projected, then simultaneously stitched and registered across imaging cycles. Lastly, the aligned stitched images are sliced again into smaller tiles for computational efficiency. (D) Overview of processing functionalities and features built into the pipeline: Packages for padlock probe design and downstream data analysis, such as nuclei segmentation, clustering, quality metrics and probabilistic cell typing functionalities, are included to empower researchers with an end-to-end ISS solution from assay design to computational analyses. Created with BioRender.com.

**Fig. 2.**
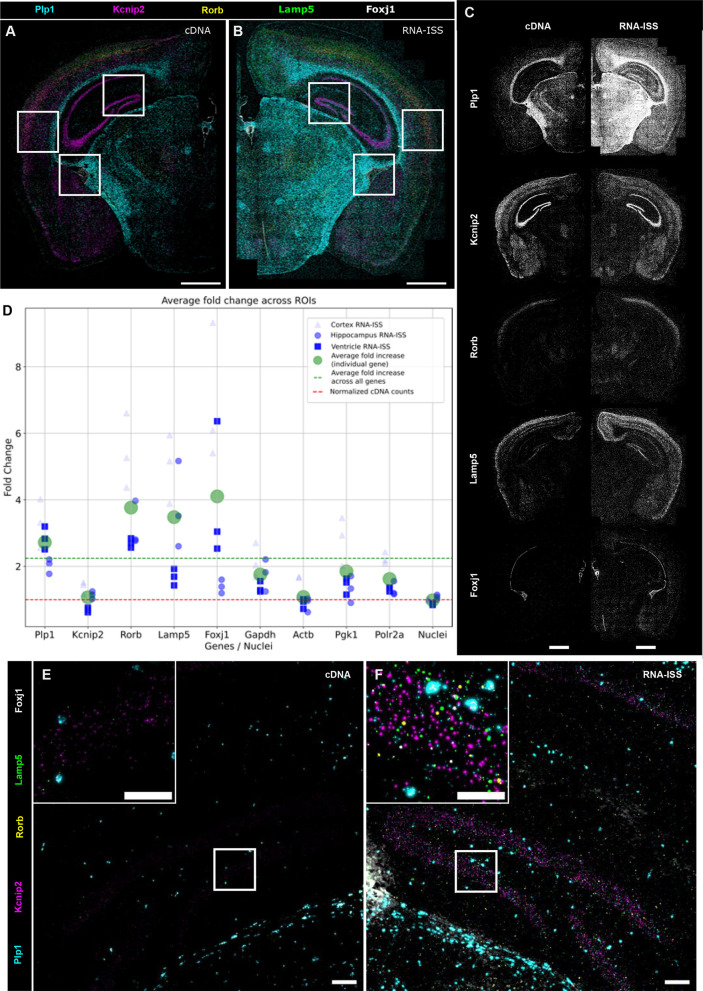
**RNA-ISS recapitulates known mRNA expression patterns with improved efficiency.** (A,B) Expression distribution of *Plp1* (cyan), *Kcnip2* (magenta), *Rorb* (yellow), *Lamp5* (green) and *Foxj1* (gray) across sequential half coronal mouse brain sections. The output is shown as a scatterplot of detected transcripts from cDNA-ISS (A) and RNA-ISS (B). Scale bars: 100 µm. (C) Individual gene expression of the 5-plex gene panel from cDNA-ISS and RNA-ISS as shown in A,B, with the output shown as a scatterplot. The 4-plex mouse reference genes (*Actb*, *Gapdh*, *Pgk1* and *Polr2a* are shown in Fig. S1E). Scale bars: 100 µm. (D) Normalized RCPs/gene counts across regions of interests (ROIs) (hippocampus, lateral ventricle and cortex; boxed regions in A,B; Fig. S1A-D) for RNA-ISS against cDNA-ISS. On average, a 2.38-fold increase in detection was achieved with RNA-ISS. *n*=3, standard deviation=1.18. (E,-F) Representative raw image of spatial distribution of 5-plex genes across one of the three ROIs (Fig. S1A-D) between cDNA-ISS (E) and RNA-ISS (F). Images are adjusted to the same contrast levels for both chemistries. Scale bars: 100 µm or 50 µm (insets).

We introduced a detection chemistry based on the direct ligation of padlock probes on their target RNA, avoiding the cDNA synthesis step (Fig. 1A,B). This chemistry uses chimeric padlock probes in combination with T4 RNA ligase2, as previously suggested for *in vitro* applications (Krzywkowski et al., 2019), to achieve higher detection efficiency and a simplified workflow. We profiled a panel of nine genes on consecutive mouse coronal brain sections with cDNA-ISS and RNA-ISS, using the same number of probes per transcript across protocols. These genes are expressed in different cell types such as oligodendrocytes (*Plp1*), excitatory neurons (*Rorb* and *Lamp5*), inhibitory neurons (*Kcnip2*) and epithelial cells (*Foxj1*) and have known unique spatial expression patterns, and also include a set of housekeeping genes (*Actb*, *Gapdh*, *Pgk1* and *Polr2a*), as described in Gyllborg et al. (2020) (Fig. 2A; Fig. S1).

RNA-ISS recapitulates the spatial expression of all genes (Fig. 2B,E,F), with an average sensitivity increase of 2.38-fold over cDNA-ISS (Fig. 2C,D, standard deviation=1.18), implying a set of potential advantages. First, more fine-grained information can be extracted from the same tissue, allowing the resolution of a higher number of transcripts per cell. Second, genes with lower expression levels can be more efficiently detected. Third, informative signal density might be extracted with a reduced set of probes. A careful analysis of the detected signal spots across methods revealed that RNA-ISS captured a higher number of spots outside the tissue compared with cDNA-ISS (Fig. 2C; Fig. S1E), prompting the need to rule out technical artifacts. The higher sensitivity of RNA-ISS does not appear to be explained by this increased detection outside the tissue (Pearson correlation=−0.39, *P*=0.29), but does instead correlate with the RNA/cDNA ratios inside the tissue (Pearson correlation=0.99, *P*=5×10^−15^). This suggests that reads outside of the tissue have a minor impact on the fold-change increase. Furthermore, RNA-ISS counts outside the tissue were correlated with the expression levels inside the tissue (as detected by cDNA-ISS) (Pearson correlation=0.88, *P*=0.001), suggesting that these spots could be mRNA molecules smeared over the glass during sample handling and are not artifacts generated by a spurious activity of the ligase.

When correcting the fold change for the presence of spots outside the tissue, RNA-ISS was still twice as efficient as cDNA-ISS (average=1.96, standard deviation=1.04). From this comparison, we estimated the capture efficiency of RNA-ISS to be about 2-10%, against 1-5% for cDNA-ISS (Magoulopoulou et al., 2023). To place these values in a common reference frame, single-molecule fluorescence *in situ* hybridization (smFISH) has a capture efficiency of over 90%, whereas droplet-based single-cell RNA-sequencing (scRNAseq) efficiency is around 30%, according to the 10× Chromium manual, which is roughly comparable with the efficiency of 10× Xenium (Salas et al., 2023 preprint).

We validated the robustness of the RNA-ISS by applying it to several animal model species: *Drosophila melanogaster*, *Mus musculus* and *Gallus gallus*, with essentially no modifications to the protocol. On fly ovaries, we probed for a set of germline and somatic mRNAs. As expected, *vasa* (also known as *vas*) and *nanos* mRNAs were detected exclusively in the nurse cells and in the oocyte (germline), *traffic jam* (*tj*) expression was exclusive to the follicular epithelium (somatic), whereas *slow border cells* (*slbo*) expression was exclusive to a subgroup of somatic cells called border cells (Fig. S2A-G). The perinuclear distribution of *gurken* mRNAs was also correctly resolved, indicating that our method accurately preserves the subcellular localization of mRNAs (Fig. S2D). We then further probed genes expressed in mouse (15 genes) and chicken (64 genes) brains and computationally decoded their expression. For both species, the decoded expression patterns were localized, specific and consistent with previous available knowledge (see Fig. S2H-K for chicken gene expression patterns, and data at https://lee2024supp.serve.scilifelab.se/ for both chicken and mouse gene expression patterns).

### RNA-based ISS is compatible with standard labeling techniques

**Fig. 3.**
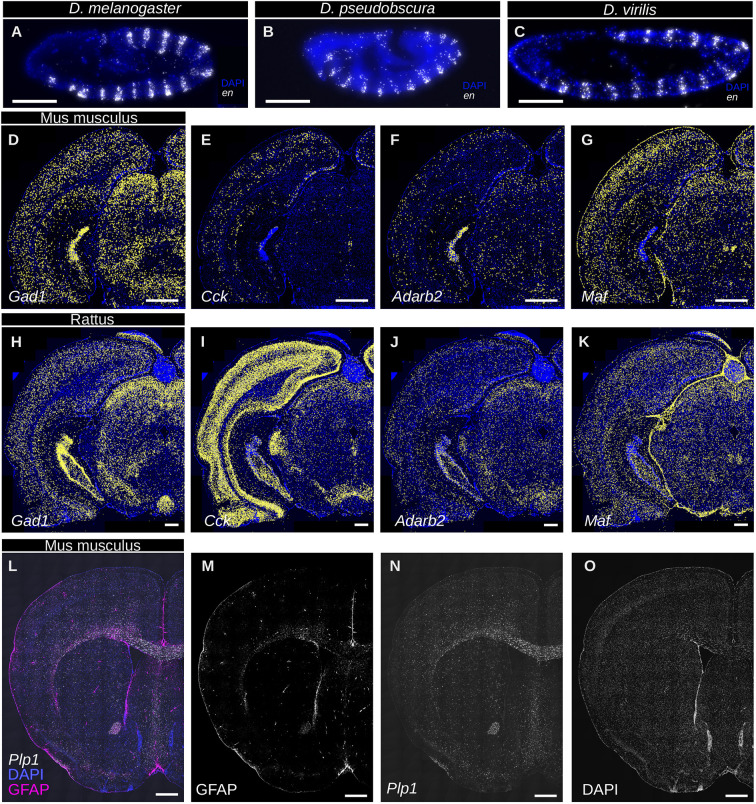
**RNA-ISS enables cheap comparative analysis in related species and is compatible with antibody labeling.** (A-C) Probes targeting conserved regions detect the mRNA of *engrailed* (*en*) across multiple related species, i.e. *Drosophila melanogaster* (A), *Drosophila pseudobscura* (B) and *Drosophila virilis* (C), with comparable efficiency. The species cover an evolutionary divergence time of approximately 40 million years. Scale bar: 10 µm. (D-K) Probes targeting conserved regions allow detection in mouse and rat. The same genes (*Gad1*, *Cck*, *Adarb2* and *Maf*) were probed on mouse (D-G) and rat (H-K) brains using the probes designed against mouse genes, showing similar expression patterns. *Gad1* (D,H), a GABAergic neuron marker, is expressed by inhibitory neurons in the cortex, hippocampus, thalamus and superior colliculus, as well as in the midbrain tegmentum. *Cck* (E,I) and *Adarb2* (F-J), both interneuronal markers, label interneurons in the cortex and hippocampus. *Adarb2* also shows enriched expression in the telencephalic choroid plexus, and scattered expression in the midbrain. *Maf* (G-K), an inhibitory neuronal marker, is expressed in the cortex, hippocampus, superior colliculus and thalamus, and highly enriched expression appears in the periaqueductal grey matter, Scale bars: 1 mm. (L-O) RNA-ISS can be combined with posterior (or simultaneous) immunohistochemistry. After performing ISS, the detection probes were stripped and the tissue stained using an antibody against the GFAP protein (M). The expression of the *Plp1* mRNA was also re-labeled with a fluorescence detection oligonucleotide (N), to provide a contrast reference. DAPI counterstaining is shown (L,O). Scale bars: 1 mm. Images are representative of two animals for mouse and rat experiments, and ten embryos for each *Drosophila* species.

Combining routine assays with ISS might be of crucial interest to a wider scientific community. To test whether ISS could be combined with immunostaining, after the last cycle of ISS on mouse brains, we stripped all the detection probes and proceeded to stain against the GFAP protein, successfully recapitulating the expected specific pattern (Fig. 3L-O). This is of potential value for different reasons: (1) it allows researchers to correlate ISS data with known landmarks on the tissues, marked by the specific expression of a given protein; (2) in samples for which multiplexed antibody panels are available (e.g. human), high-plex multi-modal interrogation of a tissue might be possible; (3) simultaneous analysis of mRNA and proteins encoded by the same set of genes potentially allows the study of mRNA translation dynamics; and, finally, (4) staining for membrane proteins might improve transcript segmentation. We demonstrate an example of the second point by performing multiplexed antibody stainings post RNA-ISS using co-detection by indexing (CODEX). We ran RNA-ISS on a mouse coronal section with a previously described probe panel (Gyllborg et al., 2020; Ke et al., 2013; Lee et al., 2022) and posteriorly labeled the tissue with 11 barcoded antibodies [labeling the proteins ACTB, CD31 (encoded by *Pecam1*), CNP, GFAP, KI67 (encoded by *Mki67*), LMNB1, MOG, MRC1, NEFL, S100B and synaptophysin (encoded by *Syp*)], generally recapitulating both the expected gene expression patterns and protein distribution. For some of the antibodies we could detect, together with the specific labeling, non-specific staining in the corpus callosum, as illustrated in Fig. S3. This background staining appears to arise as a consequence of the DNA conjugation to some antibodies, and it is not specifically produced by the combined CODEX+ISS workflow (see Materials and Methods, ‘Antibody conjugation’ section). This suggests a broad compatibility of RNA-ISS with downstream multiplexed antibody stainings, potentially enabling multi-omics approaches (https://lee2024supp.serve.scilifelab.se/mouse_ISS_CODEX.tmap).

We then finally tested the possibility of combining ISS with 5-ethynyl-2′-deoxyuridine (EdU)-labeling for simultaneous gene expression and birth dating analysis: the results from these experiments, shown in the companion paper by Rueda-Alaña et al. (2024), indicate that ISS- and EdU-based birth dating can be easily combined on the same tissue sample, allowing the interrogation of both gene expression and timing of origin of selected cell types within a tissue.

These two examples of how ISS can be integrated with existing techniques showcase only a relatively small range of potential applications. Other ideas that come to mind include the combined use of ISS with genetically encoded photoconvertible sensors (e.g. CaMPARI; Fosque et al., 2015) or with clonal tracing tools such as Cre-Lox (Sauer and Henderson, 1988) or FLP-FRT (Golic and Lindquist, 1989). Intuitively, in order to avoid interference between reporter fluorescence and the ISS readout, this type of experiment requires adjustments to the experimental and imaging protocols. We discuss some of our thoughts and experiences in the ‘Technical notes and considerations for successful ISS experiments’ section of the supplementary Materials and Methods.

### Cross-reactive design of probes allows low-cost comparative analysis of gene expression

A peculiar feature of the RNA-based ISS chemistry is its mismatch-tolerance. Although the direct probing of RNA produces an overall increase in detection efficiency, it also generates a small specificity cost, because the enzyme of choice is, to some extent, mismatch tolerant (Krzywkowski et al., 2019). Our pipeline includes a stringent specificity check to prevent the design of probes with predicted off targets based on sequence similarity (see Materials and Methods). As a result of this check, it is sometimes impossible to design padlock probes to discriminate among closely related transcripts (e.g. recent gene duplications), because their sequence has not diverged sufficiently to fall above the ligase specificity threshold.

We sought to turn this limitation into an advantage, rationally designing cross-reactive probes to recognize the mRNA of the same gene across multiple related species, thus enabling comparative analysis of gene expression with a reduced cost (Tables S7, S8, S10). We tested this by designing a set of padlock probes against the *engrailed* (*en*) gene of *D. melanogaster*, explicitly selecting targets with a high sequence conservation. Besides *D. melanogaster*, this probe set shows specific and sensitive activity on *Drosophila pseudobscura* and *Drosophila virilis* embryos, showing that this design can be used to experimentally cover potentially large evolutionary periods – the last common ancestor of *D. melanogaster* and *D. virilis* is estimated to be 40 million years ago (Nurminsky et al., 1996), a time interval corresponding roughly to the split between old- and new-world monkeys (35 million years ago) (Schrago and Russo, 2003) (Fig. 3A-C).

We successfully tested the same design on a set of genes (*Gad1*, *Cck*, *Adarb2* and *Maf*) expressed in mouse and rat midbrains, using a set of probes cross-reactive in both species (which are about 30 million years apart; Adkins et al., 2001) (Fig. 3D-K). Finally, we predicted *in silico* the cross-reactivity with *Macaca fascicularis* genes of a large panel of 1776 probes previously designed against 363 human genes. These probes were not explicitly designed to be cross-reactive; however, we found that roughly 74% of them are predicted to recognize specific individual genes in *M. fascicularis*, 12% are predicted not to bind to any transcript, and 14% are predicted to recognize off-target mRNAs. In practical terms, this suggests that this specific probe panel might be used to address questions in *M. fascicularis*, provided that non-binding and off-target-binding probes are excluded during library preparation. Assuming similar expression levels across species, we would expect an average drop of 25% in detection efficiency, which might be a tolerable loss, depending on the specific biological question a scientist might have. However, as suggested by the mouse-rat comparison, it is not always obvious how efficiently a set of probes might work across species, because the observed variation might be explained by a number of confusing factors (biology, presence of non-annotated off targets, RNA integrity difference). We further discuss the implications of cross-reactive design in the supplementary Materials and Methods. In any case, we believe that the rational design of cross-reactive probes is a potentially useful feature for laboratories focused on comparative work, allowing the spatial analysis of large numbers of genes across multiple related species.

### A toolbox for design and data analysis of ISS experiments

To enable naive users to design reagents for RNA-based ISS, process the imaging data and analyze the experimental results, we compiled a set of Jupyter notebooks and installable packages that can be easily followed, as well as a step-by-step manual for image preprocessing and analysis (Fig. 1B-D; supplementary Materials and Methods). We also provide a small test dataset (https://figshare.com/s/8e0c2bd43a3975fcff4a) to allow users to check the correct installation of packages and explore the various modules. We give here a brief overview of the main functionalities of each notebook, but we refer to the supplementary Materials and Methods for more detailed information and guidance.

#### Probe design

Given a set of gene identifiers and a transcriptome of reference, the software extracts a number of suitable target regions from each mRNA sequence and checks these regions for off targets in the transcriptome. The script also associates each gene to a unique barcode and assembles the final padlock probe sequences.

#### Preprocessing

This module transforms the raw images into a format suitable for decoding, executing the following steps: (1) the images are maximum *z*-projected; (2) the resulting 2D-projected images (‘tiles’) are simultaneously stitched and registered across imaging cycles, using the ASHLAR software (Muhlich et al., 2022); and (3) the aligned stitched images are sliced again into smaller tiles, to allow a faster and computationally efficient decoding.

#### Decoding

The preprocessed images are converted into SpaceTX format (Axelrod et al., 2021) and piped into the Starfish Python library (Tables S11 and S12) (https://github.com/spacetx/starfish). The output is a CSV table, with each row representing an mRNA detection event and each column showing different properties (*x* and *y* positions, corresponding gene, quality metrics, etc.). A set of functions allows the users to generate plots and figures for individual genes and to inspect some of the performance-decoding metrics.

#### Postprocessing

Several functions enable the user to segment cells using some published algorithms, such as Cellpose (Stringer et al., 2021) and Stardist (Schmidt et al., 2018), and to assign transcripts to cells and create Annotated Data objects (AnnData) for more advanced analysis using the scverse ecosystem of tools (Virshup et al., 2023).

New users wishing to repurpose an available microscope to perform ISS would essentially need to interface the output from the microscope with the provided preprocessing module. Once the preprocessing step is correctly completed, the other modules are expected to work without modification. We detail in the manual (see supplementary Materials and Methods) the requirements of the input files for the preprocessing module.

### Summary

To conclude, we provide an open-source method for high-sensitivity ISS that is readily applicable to a wide range of tissue types and animal model organisms, both vertebrates and invertebrates. We believe that this method could be easily transferred to other species (including non-animals) with minimal modification. The wet laboratory workflow is easily implemented on top of standard procedures such as immunostaining and EdU labeling. We also provide a simplified bioinformatic pipeline for data mining, decoding and advanced analysis of ISS datasets, from raw microscope images to cell clusters, as well as a complete guide to the entire analysis workflow. We hope that this work encourages the adoption of imaging-based spatial omics by a large and diverse community of scientists in the developmental biology field.

## MATERIALS AND METHODS

### Probe design, synthesis, pooling and phosphorylation

The notebook for probe design provided at https://github.com/Moldia/Lee_2023/tree/main/PLP_directRNA_design performs the following operations. Given a list of gene identifiers and a transcriptome of reference, the mRNAs corresponding to all the described isoforms of each gene are extracted and aligned using CLUSTALW2 (Larkin et al., 2007). The common regions are then sliced in all the possible 30-mers that compose them (nucleotides 1-30, 2-31, etc.), and the resulting 30-mers are filtered according to increasingly restrictive criteria to find suitable targets for the binding of the padlock probes. First, a GC content filter is applied, to retain only 30-mers within a given range. Among these, only the 30-mers with a C or G in position 16 are kept.

A random subset of non-overlapping suitable 30-mers spanning the transcript is then ‘specificity checked’: each target is searched against the transcriptome, using the pattern-matching capability of Cutadapt (Martin, 2011) and allowing for up to six mismatches, a conservative threshold under which we expect at least some degree of cross-reactivity. 30-mers showing non-specific hits (i.e. hits that do not belong to the query gene) are discarded. Because T4 RNA ligase 2 is slightly promiscuous, performing this step ensures that the padlock probes will have no off targets. Of all the 30-mers passing the specificity check, a subset (normally composed of five targets) is chosen, and a final step creates a specific padlock probe sequence in which unique barcodes are associated with unique genes.

To design explicitly cross-reactive probes for multiple fly species, we performed the 30-mer step extraction for all the *engrailed* orthologues across the 12 *Drosophila* species. We then kept for the downstream steps only the 30-mers that had a difference of less than six nucleotides from the *D. melanogaster* sequence. Finally, we filtered out all the 30-mers that could produce secondary hits in the transcriptome, using our specificity check based on Cutadapt.

Similarly, for mouse and rat cross-reactivity, we ran the 30-mer search in both species and retained only the 30-mers found in both species, with a tolerance of six mismatches. Of these, hits with potential off targets were excluded in the posterior filtering steps.

To assess the possible cross-reactivity of a human probe panel on *M. fascicularis*, we ran a specificity check for the human probes with a six-nucleotide-mismatch tolerance. We then classified the probes in three categories according to the results: (1) probes that recognize the mRNA of a single specific gene (‘cross-reactive and specific’); (2) probes that recognize the mRNAs of more than one gene (‘cross-reactive but non-specific’); and (3) probes that do not recognize any mRNA (‘non-cross-reactive’).

The probes were ordered as RNA Ultramers with a 3ʹ terminal RNA base from Integrated DNA Technologies, with a synthesis scale of 4 nmol, resuspended in IDTE buffer at a 200 μM concentration.

Probes can be either ordered pre-phosphorylated (with some added cost) or phosphorylated in house. When working with large pools, we used the latter approach. We pooled all the padlock probes for a given experiment in a single tube, and performed phosphorylation according to the following protocol: 20 nmol of the pooled probes were phosphorylated in 1× PNK buffer (B0201S, New England Biolabs), 1 mM ATP (P0756S, New England Biolabs) and 20 U of T4 Polynucleotide kinase (M0201L, New England Biolabs) in a total volume of 50 µl for 2 h at 37°C. PNK was then heat inactivated at 65°C for 5 min and the pooled phosphorylated probes were stored at −20°C until further use.

### Tissue fixation and pre-processing

All animal experiments were approved by the University of the Basque Country (UPV/EHU) Ethics Committee (Leioa, Spain) and the Diputación Foral de Bizkaia, and conducted in accordance with personal and project licenses in compliance with the current normative standards of the European Union (Directive 2010/63/EU) and the Spanish Government (Royal Decrees 1201/2005 and 53/2013, Law 32/107). Fertilized chick eggs (*Gallus gallus*) were purchased from Granja Santa Isabel (Córdoba, Spain). They were incubated at 37.5°C in a humidified atmosphere until the required developmental stage. The day when eggs were incubated was considered embryonic day (E) 0. Chicken (E15) brains were dissected and immediately immersed in OCT embedding medium (361603E, VWR) for a washing step, and then transferred to an embedding mold containing OCT for embedding and freezing. The embedding mold was transferred to a dry-ice box and frozen for 10 min (we found that this freezing method produces better tissue integrity than liquid nitrogen). Samples were stored at −80°C until sectioning. Embedded tissue blocks were sectioned on a CM1950 cryostat (Leica Microsystems) in slices 10-20 µm thick and attached to SuperFrost Plus microscope slides (631-0108, VWR). The sections could be stored at −80°C indefinitely. The first day of the library preparation protocol, we thawed the samples for 5 min, allowing them to reach room temperature (RT), washed the slides in PBS and immersed them in 3% paraformaldehyde (PFA) for 5 min. This is the starting point of the library preparation protocol referred below when working with fresh-frozen material.

For *Drosophila* tissues, we collected *yw* overnight egglays on apple juice agar plates, dechorionated the embryos with bleach, and fixed them in a 1:1 mix of 4% formaldehyde in PBS and heptane. We then removed the heptane, devitellinized the embryos using methanol, and stored them until the day of embedding. Similarly, ovaries were dissected on ice-cold PBS and immediately fixed in 4% formaldehyde for 20 min, then washed with PBS three times, dehydrated progressively in methanol:PBS (1:3, 2:2, 3:1, 20 min each), washed twice in methanol and stored in methanol at −20°C until embedding.

For embedding fly tissues, we rehydrated them progressively in methanol:PBS (3:1, 2:2, 1:3, 20 min each) and washed twice in PBS. We then cryoprotected the tissues overnight with 30% sucrose in PBS, transferred the tissues to OCT-containing embedding molds, and froze the samples on dry ice for 10 min. Samples were stored at −80°C until sectioning. Embedded tissue blocks were sectioned on the Leica CM1950 cryostat in slices 10-20 µm thick and attached to SuperFrost Plus microscope slides. The sections could be stored at −80°C indefinitely. On the first day of the library preparation protocol, we thawed the samples for 5 min, allowing them to reach RT, and washed them with PBS. This is the starting point of the library preparation protocol referred below when working with PFA-fixed material.

Rat and mouse brain slices were purchased fresh frozen (Zyagen, RF-201-10, strain SD and MF-201-08, strain CD1, respectively), kept at −80°C until use and processed the same way as chicken sections.

### cDNA-ISS library preparation

The protocol was followed as that published previously (Gyllborg et al., 2020) and available at protocols.io (https://doi.org/10.17504/protocols.io.xy4fpyw).

### RNA-ISS library preparation

Fixed slides were washed three times with PBS at RT and permeabilized with a 0.1 N HCl incubation for 5 min, followed by two washes in PBS. The samples were progressively dehydrated with a 70% ethanol bath for 2 min, followed by a 100% ethanol bath for 2 min, then air dried. We attached secure-seal chambers to cover the samples, and filled the chamber with PBS containing 0.5% Tween 20, followed by a PBS wash.

A probe solution was prepared according to the following recipe: 2× SSC (Invitrogen, AM9765), 10% formamide and 10 nm of each padlock probe. The samples were then incubated with the probe solution overnight at 37°C (Tables S1-S3). The next day, we washed the unhybridized excess probes by two washes of 10% formamide in 2× SSC, followed by two washes in 2× SSC. After removing the last SSC wash, a ligation mix was prepared as described in the ‘Probe ligation’ step in our ‘Home made direct RNA detection’ protocol (https://www.protocols.io/view/home-made-direct-rna-detection-kqdg39w7zg25/v1) and incubated on the samples for 2 h at 37°C. After ligation, the samples were washed twice with PBS and an amplification mix was prepared as in the ‘Amplification of the padlock probes’ step in the above protocol. The amplification reaction was carried out overnight at 30°C. The next day, the samples were washed three times with PBS and L-probes (or bridge probes) were incubated for 30 min as described in the step ‘Sequence By Hybridization: Bridge-Probe and Detection Oligo’ in the HybISS protocol (https://www.protocols.io/view/hybiss-hybridization-based-in-situ-sequencing-kqdg34357l25/v1). Excess probes were washed out with two washes in 2× SSC and detection oligonucleotides and DAPI were incubated for 30 min as indicated in the HybISS protocol. Excess detection oligonucleotides were washed out with two washes in 2× SSC. If necessary, TrueBlack (Biotium) was applied to quench background fluorescence, according to the manufacturer's instructions. In the present work, Trueblack was only applied to the chicken optic tectum slides. Samples were mounted in SlowFade gold, and cyclical imaging was performed. After each cycle of imaging, L-probes and detection oligonucleotides were stripped with two washes for 3 min each in 100% formamide, followed by five washes in 2× SSC. Hybridization of L-probes and detection oligonucleotides for the following detection cycle was performed as above (Tables S5 and S6).

### cDNA-ISS and RNA-ISS comparison

Nine genes were selected for the efficiency benchmarking comparison. A panel of five genes (*Plp1*, *Lamp5*, *Rorb*, *Foxj1* and *Kcnip2*; five probes per gene) was selected for their specificity in marking different cell types (oligodendrocytes, excitatory and inhibitory neurons and ependymal cells) with unique spatial expression patterns. An additional panel of four mouse reference genes (*Gapdh*, *Actb*, *Pgk1* and *Polr2a*; four probes per gene) as described in Gyllborg et al. (2020) was supplemented to the 9-plex gene panel (Tables S1 and S4). Briefly, post library preparation and fluorescence labeling of genes of interest, rolling circle product counts for the respective genes were acquired using our preprocessing and decoding modules, quality filtered, quantified across the half coronal mouse brain, and were used for the comparison of detection efficiency of the respective chemistries. The images were acquired on consecutive sections, using the same microscope and unchanged imaging settings, and analyzed using the same image analysis pipeline with identical detection criteria.

### Image acquisition for RNA-ISS

Imaging was performed using a standard epifluorescence microscope (Leica DMI6000) connected to an external LED source (Lumencor SPECTRA X light engine). The light engine was set up with filter paddles (395/25, 438/29, 470/24, 555/28, 635/22, 730/40). Images were obtained with a sCMOS camera (2048×2048, 16-bit, Leica DFC90000GTC-VSC10726), automatic multi-slide stage, and 20× (HC PL APO 20×/0.80 DRY, 11506529) and 40× (HC PL APO 40×/1.10 WATER, 11506342) Leica Apochromat objectives. The microscope was equipped with filter cubes for six-dye separation (Alexa Fluor 750, Cy5, Cy3, Alexa Fluor 488, Atto425 and DAPI) and an external filter wheel (DFT51011).

Each region of interest (ROI) was marked and saved in the Leica LASX software for repeated imaging. Each ROI was automatically subdivided into tiles. For 40× imaging of each tile, a *z*-stack with an interval of 0.5 μm was acquired in all the channels. For 20× imaging of each tile, a *z*-stack with an interval of 1.0 μm was acquired in all the channels. The tiles were defined to have a 10% overlap at the edges. The images were saved as thousands of individual TIFF files with associated metadata.

### Anti-GFAP antibody staining

After RNA-ISS detection, the tissue was washed three times in 100% formamide for 2 min to remove hybridized detection probes, then washed five times in PBS. Next, the tissue was blocked with PBS containing 5% normal donkey serum (Jackson ImmunoResearch) and 0.5% Triton X-100 for 1 h. Sections were incubated with primary antibody against GFAP (Dako, Z0334, 1:250) overnight at 4°C. Sections were then washed three times with PBS and incubated with secondary antibodies (Alexa Fluor 488 anti-rabbit IgG, Invitrogen, A-21206, 1:500) for 2 h at RT and counterstained with DAPI.

### CODEX protocol

#### Antibody conjugation

The 11-plex panel (Table S9) consisted of both pre-conjugated antibodies (*n*=2, Akoya Biosciences) and other commercially available antibodies (*n*=9). For conjugation, the protocol from Akoya Biosciences was used. First, filters (50 kDa molecular mass cut off) were blocked with 500 μl filter-blocking solution (Akoya Biosciences, 7000009). For each conjugation, 50 μg of antibody was used and transferred on blocked filters. Following the transfer, the filters were centrifuged at 12,000 ***g*** for 8 min. Then, the reduction solution (Akoya Biosciences, 7000009) was added on the filters and incubated for 30 min at RT. The reduction solution was removed by centrifugation at 12,000 ***g***. After the removal of the reduction solution, conjugation solution was added (Akoya Biosciences, 7000009). The barcodes were hydrated with 10 μl nuclease-free water (Lifer Technologies, AM9937) and 210 μl of conjugation solution. The hydrated barcodes were added on the reduced antibodies and incubated for 2 h at RT. The conjugation solution was removed via centrifugation at 12,000 ***g*** and purification solution (Akoya Biosciences, 7000009) was added to the columns. Then, 100 μl of antibody storage buffer (Akoya Biosciences, 7000009) was added to the columns and they were centrifuged at 3000 ***g*** for 2 min.

The specificity of the conjugated antibodies was checked by manual incubation of CODEX reporters. The tissue samples were incubated with a screening buffer containing 10× CODEX buffer (Akoya Biosciences, 7000001), nuclease-free water and DMSO (Sigma-Aldrich, 472301). After incubation, a reporter stock solution was prepared with screening buffer, assay reagent (Akoya Biosciences, 7000002) and nuclear stain (Akoya Biosciences, 7000003). Then, 2.5 μl from each reporter was added to the reporter stock solution and 100 μl from the prepared reporter solution was incubated with the tissue samples for 5 min in the dark at RT. The screening buffer was used for the removal of excess reporters after the incubation and the tissue samples were mounted with the Fluoroshield mounting medium (Invitrogen, 00495802).

In a few cases, we could detect some background autofluorescence in the white matter (specifically in the corpus callosum), arising after DNA conjugation to the antibodies. In these cases, we chose to use the antibodies whenever they retained highly specific labeling in other regions of the brain. Examples for these are CD31, LMNB1 and KI67.

#### Immunostaining for CODEX imaging post RNA-ISS

Post library preparation of RNA-ISS, the tissue was incubated in hydration buffer (Akoya Biosciences, 7000008) for 2 min. For tissue equilibration, the tissue was incubated in the staining buffer (Akoya Biosciences, 7000008) for 30 min. After equilibration, tissue blocking was performed using the primary antibodies (dilutions given in Table S9), which were diluted in the blocking buffer (Akoya Biosciences, 7000008), and the tissue was incubated for 3 h inside a dark humidity chamber at RT. The tissue was washed with a staining buffer for 2 min and fixed with 1.6% PFA diluted in a storage buffer (Akoya Biosciences, 7000008) for 10 min. The tissue was washed with PBS, incubated in ice-cold (4°C) methanol (Sigma-Aldrich, 322415) for 5 min and washed again with PBS. As the last fixation step, the tissue was fixed with fixative reagent (Akoya Biosciences, 7000008) and washed with PBS, before storage at 4°C.

#### Image acquisition via the CODEX system

Reporter probes were diluted in reporter stock solution in nuclease-free water (Life Technologies, AM9937), 10× CODEX buffer, assay reagent and nuclear stain reagent. Diluted reporters were placed into the corresponding wells in 96-well plates according to the experimental plan.

Automated imaging was performed using a CODEX system integrated with a Leica DMI8 microscope (Leica Microsystems, 11090148013000). The microscope had a SOLA light engine light source (Lumencor, 16740), equipped with a Leica HC PL APO CS2 20× objective, a Hamamatsu camera (2048×2048, 16-bit, C13440-20C-CL-301201) and an automated stage (ITK Hydra XY). For signal detection, we used the following Chroma filters: QUAD-S filter set: DFTC (DC:425; 505; 575) and Y7 filter (DC:760). Imaging was performed via the LASX software (Leica Microsystems), following the instructions of the CODEX software. For the selected ROI, manual focus points were located by selecting 9 as the *z*-step number. The background subtraction, deconvolution, extended depth of field and shading correction were performed on the output images using the CODEX Processor 1.7.0.6.

Post image acquisition via the CODEX system, the sample was labeled with L-probes and detection oligonucleotides and imaged as described in the RNA-ISS protocol above.

#### Alignment of images from RNA-ISS and the CODEX system

The images acquired from the CODEX system for antibody staining and RNA-ISS images imaged on the Leica DMI6000 microscope (as documented above) were first roughly aligned with the affinder plugin on Napari (https://www.napari-hub.org/plugins/affinder), yielding a first transformation matrix that allowed for a first alignment. Next, for a close to pixel-perfect alignment, the images were aligned for a second time with scipy.optimize from Elegant SciPy (https://github.com/elegant-scipy/notebooks/blob/c7f4cc84deaceb132cf697ae359e75ff4881590b/notebooks/ch7.ipynb).

### Data processing

The raw images and the associated metadata from the microscopes were fed into the preprocessing module of our analysis pipeline (https://github.com/Moldia/Lee_2023/tree/main/ISS_preprocessing). The module transforms the images into a format suitable for decoding, executing the following steps. First, the images are maximum *z*-projected. The resulting 2D projected images (‘tiles’) are simultaneously stitched and registered across imaging cycles, using the ASHLAR software (Muhlich et al., 2022). ASHLAR captures the metadata and places the tiles correctly in the *xy* space before starting the alignment step. During the process, the 10% overlap is also removed to produce stitched images. Finally, the aligned stitched images are sliced again into smaller tiles, to allow a faster and computationally efficient decoding. The resliced aligned images are then taken over by the decoding module (https://github.com/Moldia/Lee_2023/tree/main/ISS_decoding), which converts them into the SpaceTX format (Axelrod et al., 2021) and pipes them into the Starfish Python library for decoding of image-based spatial transcriptomics datasets (https://github.com/spacetx/starfish). Within the decoding modules, the images are normalized across channels and imaging cycles, a spot detection step is performed, and for each detected spot, its intensity across all channels is extracted. For each spot, the prominent channel for each cycle is extracted and a spot identity is annotated in color space. Each spot is now represented by a sequence of colors across cycles. The color sequence of each spot is matched to a decoding table that associates a color sequence with a specific gene. The output of this decoding is a CSV table, in which each row represents a detection spot with different properties (*x* and *y* positions, gene identity and quality metrics). The quality metrics for each spot are computed as follows. For each spot, the normalized fluorescence intensities across all channels are extracted. The prominent channel is considered the ‘true signal’, and all the others are considered ‘background’. The score is described by the formula ‘true signal’/(‘true signal’+‘background’), and it has a theoretical maximum value of 1 (perfect decoding) and a theoretical minimum value of 0.25 when decoding in four colors, which corresponds to a random assignment (i.e. all the channels have the same fluorescence intensity for that spot). The quality score for each spot is computed per cycle, allowing to calculate two parameters: (1) the average quality across all cycles and (2) the minimum quality across cycles. We found that filtering according to a minimum quality produces more reliable data, and we normally used a filter value of 0.5.

For the chicken optic tectum sections, we additionally performed image deconvolution using flowdec (Czech et al., 2019) on the raw images before proceeding to the preprocessing steps. We found this step to drastically increase the number of detected spots in dense datasets.

Other modules in the repository allow users to perform additional operations on the images, and are documented in the supplementary Materials and Methods.

### Code availability

All the code used in this paper is available at https://github.com/Moldia/Lee_2023.

## Supplementary Material



10.1242/develop.202448_sup1 Supplementary information

Table S1. (M. musculus) RNA-ISS PLP sequences and corresponding LbarIDs and RCA Primer Chimeric PLP sequences for all *M. musculus* experiments with the corresponding LbarIDs and universal RCA primer sequence.

Table S2. (G. gallus) RNA-ISS PLP sequences and corresponding LbarIDs Chimeric PLP sequences for all *G. gallus* experiments with the corresponding LbarIDs.

Table S3. (M. musculus - R. norvegicus) cross reactive species RNA-ISS PLP sequences, corresponding LbarIDs and sequence similarity Chimeric PLP sequences for *M. musculus / R. norvegicus* experiments with corresponding LbarIDs and sequence similarity

Table S4. (M. musculus) cDNA-ISS PLP sequences and corresponding LbarIDs DNA PLP sequences for cDNA-ISS used in the 9plex RNA-ISS - cDNA-ISS efficiency comparison

Table S5. Lprobe library The sequences of the Lprobes used, with the corresponding detection oligo and LbarIDs

Table S11. Codebook for *M. musculus / R. norvegicus* cross reactivity experiment Codebook used for the combinatorial decoding of the cross reactivity experiment involving *M. musculus / R. norvegicus*.

Table S12. Codebook for *G. Gallus* optic tectum experiment Codebook used for the combinatorial decoding of the *G. Gallus* optic tectum experiment.
