# Supplementary material for: Open-source, high-throughput targeted in situ transcriptomics for developmental and tissue biology
Source: Development. 2024 Aug 29;151(16):dev202448. doi: 10.1242/dev.202448 (PMC11385644; doi:10.1242/dev.202448)
Supplement: Supplementary file 1 [file develop-151-202448_review_history.pdf]

Open-source, high-throughput targeted in-situ transcriptomics for developmental and tissue biology

Hower Lee, Christoffer Mattsson Langseth, Sergio Marco Salas, Sanem Sariyar, Andreas Metousis, Eneritz Rueda Alana, Christina Bekiari, Emma Lundberg, Fernando Garcia Moreno, Marco Grillo and Mats Nilsson
DOI: 10.1242/dev.202448

Editor: Debra Silver

Review timeline

Original submission:	19 October 2023
Editorial decision:	6 December 2023
First revision received:	1 May 2024
Editorial decision:	3 June 2024
Second revision received:	11 July 2024
Accepted:	19 July 2024

Original submission

First decision letter

MS ID#: DEVELOP/2023/202448

MS TITLE: Open-source, high-throughput targeted in-situ transcriptomics for developmental biologists

AUTHORS: Hower Lee, Christoffer Mattsson Langseth, Sergio Marco Salas, Andreas Metousis, Eneritz Rueda Alana, Fernando Garcia Moreno, Marco Grillo, and Mats Nilsson

I have now received all the referees' reports on the above manuscript, and have reached a decision. The referees' comments are appended below, or you can access them online: please go to BenchPress and click on the 'Manuscripts with Decisions' queue in the Author Area.

As you will see, the referees express considerable interest in your work, but have some significant criticisms and recommend a substantial revision of your manuscript before we can consider publication. If you are able to revise the manuscript along the lines suggested, I will be happy receive a revised version of the manuscript.

Amongst the major concerns, both reviewers indicate the need for stronger evidence to address the sensitivity and efficiency of ISS and why it is better than cDNA-ISS and other techniques. While Xenium analysis may not be feasible, further comparisons to existing methods and clarifications along these lines will increase the impact of your study. Please also include additional information and images to support the technique. Please also include additional methodology and address questions regarding this in your study to make it user friendly.

Your revised paper will be re-reviewed by one or more of the original referees, and acceptance of your manuscript will depend on your addressing satisfactorily the reviewers' major concerns. Please also note that Development will normally permit only one round of major revision. If it would be helpful, you are welcome to contact us to discuss your revision in greater detail. Please send us a point-by-point response indicating your plans for addressing the referees’ comments, and we will look over this and provide further guidance.

© 2024. Published by The Company of Biologists under the terms of the Creative Commons Attribution License (https://creativecommons.org/licenses/by/4.0/).

Please attend to all of the reviewers' comments and ensure that you clearly highlight all changes made in the revised manuscript. Please avoid using 'Tracked changes' in Word files as these are lost in PDF conversion. I should be grateful if you would also provide a point-by-point response detailing how you have dealt with the points raised by the reviewers in the 'Response to Reviewers' box. If you do not agree with any of their criticisms or suggestions please explain clearly why this is so.

Reviewer 1

Advance summary and potential significance to field

Lee et al. introduce the use of chimeric padlocks for in situ sequencing spatial transcriptomics. Chimeric padlocks bind directly to the RNA, skipping the cDNA synthesis and improving the sensitivity of the method. In general, they show 1) that RNA-ISS performs well in different species with minor modifications in the protocol, 2) the method is compatible with immunolabeling and EdU labelling, 3) a comprehensive pipeline for ISS data analysis. The gain in sensitivity by ~2-fold is one of the major (if not the most) advantages of this technology compared to the cDNA-based method. Since this parameter is one of the key elements to be considered when choosing a spatial transcriptomic method, and one of the most challenging to improve, I would consider the article for publication in Development. However, the authors need to provide more data supporting the improvement in sensitivity. Please, see below some considerations.

Comments for the author

Essential revisions
1) Efficiency of RNA-ISS: The authors present RNA-ISS that use chimeric padlocks and T4RNALigase2 to obtain ~2-fold increase in sensitivity in ISS.
This seems to be one of the major contributions of the article considering their previously described HybISS approach based on cDNA (Gyllborg et al NAR 2020).
However, the evidence for this key result needs to be more extensive and part of the main text/figures of the article. The Supplementary table 1 does not suffice to convince the readers of the results. I suggest including more genes (~10) in the comparison with different levels of gene expression and some images of cDNA vs dsRNA ISS. Moreover, the authors assert potential advantages of the method like a 1) higher number of transcripts per cell 2) genes with lower expression levels can be more efficiently detected 3) informative signal density can likely be extracted with a reduced set of probes, which might be useful for particular applications (detection of short mRNAs, isoform discrimination, etc.). Points 1 and 2 can be addressed with the suggestion above. It will be interesting if the authors show the performance of RNA-ISS vs cDNA in short mRNAs or isoform discrimination. Finally, since ISS is a single cell resolution method, a zoom in of the large images of the tissues will be helpful when comparing cDNA vs RNA-ISS.

2) Supplementary information: The authors didn’t include information regarding chimeric padlock sequences, L-probes, detection oligos, codebooks etc. Also, the authors showed the gene expression patterns for 4 genes (out of 15) in the mouse coronal sections, and 4 genes (out of 35) for chicken optic tectum. I understand the data is shown in the accompanying paper (Eneritz Rueda-Alaña, et al. BirthSeq, a new method to isolate and analyze dated cells from any tissue in vertebrates). However, the sequences and rest of the images need to be included as supplementary data. Finally, a side-by-side comparison with an atlas (ABA, Geisha Arizona database) will help to understand the specificity of the gene patterns.

Minor revisions
1) The authors applied RNA-ISS in a single fluorescent detection oligo manner to ‘disentangle potential artifacts of the chemistry from issues that might arise from the downstream computational decoding’. A fair comparison will be to quantify the number of RNA molecules detected using a single readout detection vs combinatorial barcoding (which needs computational decoding), and measure their correlation, either in drosophila, mouse, or chicken.

© 2024. Published by The Company of Biologists under the terms of the Creative Commons Attribution License (https://creativecommons.org/licenses/by/4.0/).

2) The authors show cross-reactive padlock probes in closely related species. It will be helpful to show as supplementary information the sequence alignment between different species and the region where the padlocks bind. Even more useful will be to include in the python pipeline the percentage of homology of the designed padlocks with the closely related species. With this, in a single run of the padlock design pipeline, it will be possible to select the probes that will work (or not) in closely related species. Regarding the mouse/rat data: some genes (e.g. Cck) seems to be highly expressed in rats compared to mice. To know if this difference is due to the species rather than the specificity to the padlock probes, please add sequencing (RNA-seq, scRNA-seq) or gene atlases data if available.

3) Either in 'RNA-ISS Library prep' or protocols.io, please provide the company and catalogue number for all the reagents used.

4) To encourage the scientific community to use RNA-ISS in a diversity of applications, it will be useful to include the cost breakdown of the method (e.g., per sample or per slide) and a comparison with the previous versions HybISS and HybRISS.

5) Finally, I would recommend using a title with a broader scope as this method can be useful beyond developmental biology.

Reviewer 2

Advance summary and potential significance to field

The authors describe an improvement to their in situ sequencing method to detect RNA transcripts in a variety of organs and organisms. First the authors introduce the use of the T4RNALigase2 to ligate the two ends of a padlock probe for rolling circle amplification. Previous iterations of the method could only ligate the ends on a DNA-DNA duplex, which first required the reverse transcription of the RNA. The reverse transcription has low efficiency and by circumventing this step, the sensitivity of the method is increased. Next, they apply the method on mouse, rat, chicken and fly tissue to demonstrate versatility of the method. Lastly, the authors demonstrate that the same probe set can be used on different but related species to detect the same gene.

In addition to the introduction of the ligase, the authors put considerable effort in sharing their method. In my opinion this is an important and timely aspect of the manuscript, because the whole field of spatially resolved transcriptomics is rapidly commercializing. Even though this trend is giving more researchers access to these technologies, the practical knowledge and know-how in academia is disappearing to these companies. With this manuscript and the online resources (protocol, guide, notebooks code), the authors enable others to build in-house versions of the technology, and flexibly adept it to their use.

Comments for the author

The authors present an improvement of the popular ISS protocol and make admirable effort to share such a complex method with protocols, instructions and code. However, the manuscript would benefit from a more thorough evaluation and comparison to other methods, to fully evaluate the advance made. I would therefore suggest the following revisions:

In my opinion the introduction of T4RNALigase2 to the ISS protocol to increase the sensitivity is the major advance of this manuscript. This builds upon the previous work of the Nilsson group (Krzywkowski et al. 2019 RNA) and follows their recent benchmark of the CARTANA kit for ISS, which also uses ligation of the padlock probe directly on the RNA (Lee et al. 2022 Scientific Reports).
Now that the CARTANA kit is no longer commercially available and the whole method is commercialized by 10X Genomics, this work provides an important resource to enable home-build ISS without commercial dependence. However, the manuscript would benefit from a more thorough validation of the protocol and detection sensitivity. Specifically:

© 2024. Published by The Company of Biologists under the terms of the Creative Commons Attribution License (https://creativecommons.org/licenses/by/4.0/).

1) The conclusion of the manuscript that RNA-ISS is better than cDNA-ISS is predominantly based on Supplementary table 1, where 3 genes in two mouse brain regions are compared. First, it would be helpful to report the standard deviation of the fold increase. Second, it is unclear why these genes are chosen and why only the thalamus and ventricle are considered in the comparison. It would be better to perform the analysis of the whole mouse brain section if possible. Furthermore, I would strongly suggest that plots of the raw data are included in the manuscript. Ideally showing the whole slide and zoomed in signal dots with nuclei segmentation outlines. In addition, as average numbers of transcript per nucleus are reported it would be good to include the number of cells in each condition. If feasible, more genes should be included to get a better estimate of the fold increase.

2) The methods indicate that tissue sections with a thickness between 10 to 20 micrometers are used for this protocol. Are the cDNA-ISS and RNA-ISS conditions performed on the same thickness? Even though transcripts per nuclei are used in the calculation, section thickness could influence the counts especially because the images are flattened with a maximum projection in Z. It would be helpful if the authors could report the number of nuclei in each section as proxy for the slice thickness.

3) Please include a protocol for the cDNA-ISS data used, or a literature reference if the dataset was already published. Also include a description of how the comparison is made in the methods.

4) To further access sensitivity between cDNA-ISS and RNA-ISS an analysis similar to Lee et al. 2022 Scientific Reports, where the distribution of counts are directly compared (Fig 1b-e) would strengthen the manuscript.
Furthermore, since many methods in this field assess their sensitivity by comparing to smFISH based data, this analysis would help place the new method in a common reference frame. Lastly, a comparison to 10X Xenium would also be interesting if possible.

The introduction, Figure 1D and online code also covers cell segmentation and clustering of ISS data. However, no demonstrations of these steps are given in the manuscript. An addition of this, for instance on the chicken data, would demonstrate the full pipeline. Because these algorithms wrap existing tools that are independently published (PCIseq, Cellpose and Scanpy) a thorough evaluation is not needed in my opinion, but an example would complete the claims made in the introduction.

Combining ISS with protein detection is a very promising capability which cannot easily be obtained with other spatially resolved transcriptomics techniques. However, in previous work the Nilsson group already demonstrated that this was possible (Mbp protein in Lee et al. 2022 Scientific reports).
Here this is again demonstrated with the GFAP protein. A stronger case would be made if multiple proteins are detected, because it is likely that not all epitopes are equally well preserved during the ISS protocol. However, this is a major effort and therefore toning down the claims would also suffice.

The authors suggest that next to EdU birth dating, other fluorescent reporters can be used in combination with ISS. However, if genetically encoded fluorescent proteins are used, will their fluorescence not interfere with the ISS signal? If they first need to be degraded would this affect the ability to use immunohistochemistry after barcode imaging?

The manuscript should better clarify to the reader that the basis for this protocol is HybISS (Gyllborg et al. NAR) rather than the original ISS protocol by Ke et al. 2013 Nature Methods.

The cross-reactive design of probes between species, is an interesting idea and can save resources in specific experimental situations where closely related species are studied. However, it also suggests that RNA-ISS could suffer from more false positives. Could the authors elaborate on this and include a measurement of false positives?

© 2024. Published by The Company of Biologists under the terms of the Creative Commons Attribution License (https://creativecommons.org/licenses/by/4.0/).

For the simulation of the Macaque probes the authors note that a human probe panel has 74% matches, 12% has no-match and 14% has off-target matches to the Macaque transcriptome. However, a discussion and conclusion lack whether that would be enough to yield high quality data.

Figure 2 A-E, why is the background so different between the images?

For none of the presented experiments the full dataset with all detected genes is shown simultaneously in a plot. Adding this would be valuable to show the alignment performance of the algorithm and give a better impression of the capabilities of the presented protocol and analysis code.

Minor comments:

The keyword “Multi-omics” is a stretch because only one protein is detected using immunohistochemistry alongside the spatial transcriptome.

Introduction:
Clarify that “sequencing-based” methods use Next Generation Sequencing, to prevent confusion with ISS.

The authors describe the rolling circle amplification products to be “large”, “very bright” and “massively amplified”. It would be more informative to specify the (approximate) size, and the amount of amplification. On a similar note, please specify what size range a “large tissue section” is.

The authors list the detection of heavily fixed mRNAs as one of the limitations of ISS, and in the next paragraph summarize that the manuscript overcomes these limitations. However, no prove (or reference to previous work) is given that indicates that over fixing is an issue. Nor, is it demonstrated that the new protocol overcomes this specific issue.

Results and discussion:

For the proof of principle experiments in Drosophila, mouse and chicken it would be helpful to see the images of the in situ reference databases side-by-side with the ISS data. A reference to the databases should also be included in the main text (Not only in the figure legend).

Include additional information on the homology between the engrailed gene in the different species. It would also be helpful to add percentages of nucleotides that differ inside the probed region between the species.

Figure 3D-G, please add a label that this is rat brain. Possibly best to do this for all species in all figures.

Material and methods:

The sequences of all probes should be available in a supplemental file.

Manufacturers all of chemicals and reagents should be included.

To which samples was TrueBlack applied?

Which objective was used to image the RCPs?

How many Z-stack were imaged?
In the filtering of spots a quality score of 0.5 is used. How is this value determined and is there any ground truth data that is used to establish this value?

ISS manual:

© 2024. Published by The Company of Biologists under the terms of the Creative Commons Attribution License (https://creativecommons.org/licenses/by/4.0/).

In the filename “TileScan 0--Stage01--C03.tif” the index after “Stage” suggests that a maximum of 100 FOVs can be included (Stage00 to Stage99). Is the algorithm limited to 100FOVs?
Please give an indication of how large a large dataset is. This will help users prepare the hardware.

Code:

The authors could consider putting a minimal raw dataset online for first time users to try.

First revision

Author response to reviewers' comments

We thank the reviewers for their constructive criticism and positive feedback. We believe their comments were very fair, and we think that the suggested revisions significantly increased the quality of our work.

To facilitate the reviewers' work, and post-peer review re-assessment of our manuscript, we deposited all the data from all the datasets mentioned in the text on the permanent viewer at: https://lee2024supp.serve.scilifelab.se/

We added this information in the Data availability section

If the reviewers and editors agree, our proposal is to cite this link in the paper as the container for all ISS supplementary data. We think this is the most intuitive way to browse all the datasets in an interactive manner, as an alternative to many pages of supplementary data showing individual gene expression patterns.

However, we also understand that permanent accessibility to these datasets is crucial for reproducibility, and we wish to reassure both the reviewers and the editors about our long term commitment: the ScilifeLab-serve hosting infrastructure will allow users to get a DOI for the published datasets by the end of the year, and we will link this information to the publication whenever it becomes available to us.

We hope this way of providing the supporting data is sufficient, but we're very open to different arrangements if the reviewers or editors consider them more appropriate (ie. supplementary figures and/or sharing the full datasets on Figshare). In the meantime, we hope these viewers at least facilitate the reviewers' task.

Here goes a point by point response to the comments:

Both reviewers suggest a more detailed comparison of RNA-ISS against the previous cDNA-based protocol. We agree this needs to be presented as a main figure in the text and supported by more extensive data.

Reviewer 1:
1) Efficiency of RNA-ISS: The authors present RNA-ISS that use chimeric padlocks and T4RNALigase2 to obtain ~2-fold increase in sensitivity in ISS. This seems to be one of the major contributions of the article considering their previously described HybISS approach based on cDNA (Gyllborg et al NAR 2020). However, the evidence for this key result needs to be more extensive and part of the main text/figures of the article. The Supplementary table 1 does not suffice to convince the readers of the results. I suggest including more genes (~10) in the comparison with different levels of gene expression and some images of cDNA vs dsRNA ISS. Moreover, the authors assert potential advantages of the method, like a 1) higher number of transcripts per cell 2) genes with lower expression levels can be more efficiently detected 3) informative signal density can likely be extracted with a reduced set of probes, which might be useful for particular applications (detection of short mRNAs, isoform discrimination, etc.). Points 1 and 2 can be addressed with the suggestion above. It will be interesting if the authors show the performance

© 2024. Published by The Company of Biologists under the terms of the Creative Commons Attribution License (https://creativecommons.org/licenses/by/4.0/).

of RNA-ISS vs cDNA in short mRNAs or isoform discrimination. Finally, since ISS is a single cell resolution method, a zoom in of the large images of the tissues will be helpful when comparing cDNA vs RNA-ISS.

As suggested by both reviewers, we increased the number of analyzed genes (9), and quantified the overall sensitivity increase on a larger tissue area, and included a plot of the number of reads/cells for all the genes across the 2 methods. From this experiment, our conclusions are:

- The average sensitivity increase of RNA-ISS vs cDNA-ISS, keeping fixed the number of probes across technologies, is in the order of 2x.
- This observed sensitivity increase is variable depending on the probed gene. While in some cases the sensitivity increase is marginal, the efficiency gain can be much higher for other genes. It's still unclear to us where this variability emerges from, but possibly further work on refining the probe design criteria will be needed to stabilize this variation.

We also added new figure panels with zoomed insets, so for readers to better appreciate the increased sensitivity at single cell resolution.

Although the idea of testing our new chemistry and comparing it with the previous cDNA iteration on the specific tasks of small RNA detection or isoform discrimination is appealing, we feel this goes beyond the scope of this work. Successful detection will be variable on a case-by-case scenario and will depend on an interplay of factors (chemistry efficiency, number of probes, expression levels). We opted to remove the claim from the main text.

2) Supplementary information: The authors didn't include information regarding chimeric padlock sequences, L-probes, detection oligos, codebooks, Etc. Also, the authors showed the gene expression patterns for 4 genes (out of 15) in the mouse coronal sections, and 4 genes (out of 35) for chicken optic tectum. I understand the data is shown in the accompanying paper (Eneritz Rueda-Alaña, et al. BirthSeq, a new method to isolate and analyze dated cells from any tissue in vertebrates). However, the sequences and rest of the images need to be included as supplementary data. Finally, a side-by-side comparison with an atlas (ABA, Geisha Arizona database) will help to understand the specificity of the gene patterns.

We apologize for the oversight. The supplementary data about probes, detection oligos, codebooks etc. is now added as supplementary information to the paper.

We also produced a supplementary figure showing the side-by-side comparison between ISS-detected genes and a sum-up scheme of previously described expression patterns in Drosophila.

Regarding the images of the mouse and rat brains, and the chicken optic data for all the genes not directly represented in the respective figures, we deposited the data into our permanent interactive TissUUmaps viewer at the link indicated above.
The data can be consulted by choosing the appropriate dataset (*'crossreactivity_mouse'*, *'crossreactivity_rat'* and *'chicken_OT'*). Alternatively they can be accessed by browsing respectively to:
https://lee2024supp.serve.scilifelab.se/crossreactivity_mouse.tmap
https://lee2024supp.serve.scilifelab.se/crossreactivity_rat.tmap
https://lee2024supp.serve.scilifelab.se/chicken_OT.tmap

Minor revisions
1) The authors applied RNA-ISS in a single fluorescent detection oligo manner to 'disentangle potential artifacts of the chemistry from issues that might arise from the downstream computational decoding'. A fair comparison will be to quantify the number of RNA molecules detected using a single readout detection vs combinatorial barcoding (which needs computational decoding), and measure their correlation, either in drosophila, mouse, or chicken.

Reviewer 1 touches here on a very important point: how much data do we lose due to

© 2024. Published by The Company of Biologists under the terms of the Creative Commons Attribution License (https://creativecommons.org/licenses/by/4.0/).

computational decoding? How does this data loss relate to the experimental settings? More crucially, can this data loss be mitigated using experimental, imaging, and data processing strategies?

On one hand, ISS is designed to be a highly multiplexed method, so computational decoding is a crucial aspect of it. On the other hand, our decoding strategy requires the detection of individual discrete signal spots, so we need imaging conditions that allow that spot detection to happen successfully.

In other words: our ability to successfully decode depends on how optically dense the images are. The implication is that our data loss caused by computational decoding is largely context-dependent, and affected by several variables: the relative density of a specific mRNA, its relative expression compared to the surrounding mRNAs, how many genes are co-decoded in the same experiment, the chosen decoding strategy etc…

A direct comparison between computational and non-computational expression analysis might therefore be only partially informative, as well as potentially misleading in some cases. We believe a more informative comparison is instead to show how computational decoding is affected by data density, under different imaging and data processing conditions. To get our point across, we'd like to share the following analysis with the reviewers.

We designed a benchmarking experiment as follows:

1) We imaged the same exact tissue area first using a 20x objective, then with a 40x objective, and performed a parallel decoding of the 2 datasets.
2) We then deconvolved both raw image sets, and repeated the decoding on the deconvolved images.
3) Finally, we applied image restoration using CARE to the projected raw images and repeated the decoding once again. This was to test the performance of CARE denoising against real deconvolution.

The hypothesis is that, if computational decoding is negatively affected by optical crowding, 20x should perform worse than 40x, and raw should perform worse than deconvolved, both in terms of absolute read count and quality metrics. The top performance is expected at high-magnification imaging followed by deconvolution.

For all the conditions we extracted 2 values, represented in the figure below. The first is the number of raw reads (light-transparent bars), while the second is the number of reads passing our internal quality threshold (solid bars), each column representing an experimental condition.

These results strongly suggest that optical crowding leads indeed to suboptimal spot detection and to a lower count of high-quality spots. Prospective users will need to critically evaluate the imaging and decoding strategy to the specific parameters of their particular experiments. We now included this analysis and its conclusions in the Supplementary Manual, under the paragraph *'Technical notes and considerations for successful ISS experiments'*.

© 2024. Published by The Company of Biologists under the terms of the Creative Commons Attribution License (https://creativecommons.org/licenses/by/4.0/).

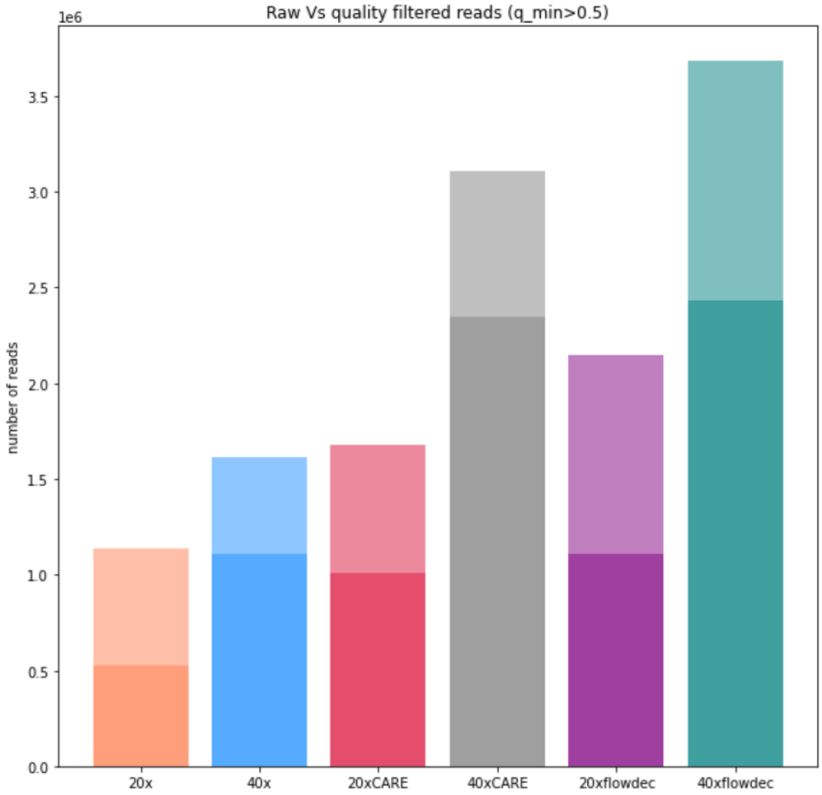

2) The authors show cross-reactive padlock probes in closely related species. It will be helpful to show as supplementary information the sequence alignment between different species and the region where the padlocks bind.

We added this information in the supplementary table.

Even more useful will be to include in the python pipeline the percentage of homology of the designed padlocks with the closely related species. With this, in a single run of the padlock design pipeline, it will be possible to select the probes that will work (or not) in closely related species.

We acknowledge that this would be a very useful function. However, designing cross-reactive probes is not only a matter of specifying a homology percentage, but also about making sure the probes are also sufficiently specific across species. One risk, in case of taxon-specific gene duplications, is that a probe designed against one gene in species A might recognise multiple paralogues in species B. Including this cross checks for multiple species in a single-run of the pipeline requires adding some specific functionalities. We are however working to set up a dash-based web app to simplify the probe design for novel users, and we'll include this functionality in it by specifying a custom workflow.

Regarding the mouse/rat data: some genes (e.g. Cck) seems to be highly expressed in rats compared to mice. To know if this difference is due to the species rather than the specificity to the padlock probes, please add sequencing (RNA-seq, scRNA- seq) or gene atlases data if available.

This result was very puzzling also to us, especially considering that the probes were designed to have perfect matches on mouse, and allow a few mismatches on rat. More intriguingly, often we have cases where we could design 5 probes for mouse, but only a subset of those are predicted to cross-react in Rat. If anything, one would have expected the probes to be more sensitive in the former rather than in the latter. Unless, of course, the increased signal in Rat is produced by spurious binding to unwanted targets, as hinted by Reviewer 1. Upon checking once again the predicted specificity of the probes via multiple means (using the pattern matching function of cutadapt, as in our probe design pipeline, or via BLAST), we have confirmed that these probes

© 2024. Published by The Company of Biologists under the terms of the Creative Commons Attribution License (https://creativecommons.org/licenses/by/4.0/).

should not have detectable off-targets in Rat, at least using the currently available transcriptomic information. We acknowledge, however, that the quality of transcriptomic annotation might be lower in the case of Rat compared Mouse. We couldn't find informative datasets to confirm our finding, and we are not sure having them would have resolved our (and the reviewer's) doubts. We speculate that the difference in detection abundance might arise because of one or more of several factors:

- The first factor is the number of cells. A rat brain is considerably larger than a mouse one, so the total count of detections is expected to be higher for that reason, assuming equal expression at the single cell level. To some extent this seems to be one of the sources of variation.
- However, zooming in on individual cells in the 2 datasets shows that clearly the Rat data looks more dense than the mouse one also at the single cell level (a very clear example is Cck), so the number of cells doesn't seem the only reason behind this difference.
- The fact that the expression patterns look very specific would suggest that the probes are working as they should. Specificity-wise, a possibility we can't account for is that these probes recognise non-annotated transcripts that were not captured by our specificity check, or by our more recent second check via BLAST. If this is happening, we have no way to know, acknowledging that working with incompletely annotated transcriptomes is tricky and might produce misleading results.
- We also speculate that, besides real differences in the gene expression levels across the 2 species, a potential cause of this difference could also be the fact that these samples might have had a different RNA quality to begin with.

We incorporated these discussion points in the section *'Technical notes and considerations for successful ISS experiments'* of the Supplementary Manual.

3) Either in 'RNA-ISS Library prep' or protocols.io, please provide the company and catalogue number for all the reagents used.

We apologize for the lack of clarity in our initial submission. The company, product name and catalog number of reagents are now documented on protocols.io as well as in the supplementary table.

4) To encourage the scientific community to use RNA-ISS in a diversity of applications, it will be useful to include the cost breakdown of the method (e.g., per sample or per slide) and a comparison with the previous versions HybISS and HybRISS.

This is a very fair point, and a very useful information for prospective users. The cost breakdown per 100ul reaction for library preparation is now documented in the supplementary table.

5) Finally, I would recommend using a title with a broader scope as this method can be useful beyond developmental biology.

We thank the reviewer for the suggestion. The new proposed title is:
"Open-source, high-throughput targeted in-situ transcriptomics for developmental and tissue biology"

Reviewer 2:
1) The conclusion of the manuscript that RNA-ISS is better than cDNA-ISS is predominantly based on Supplementary table 1, where 3 genes in two mouse brain regions are compared. First, it would be helpful to report the standard deviation of the fold increase. Second, it is unclear why these genes are chosen and why only the thalamus and ventricle are considered in the comparison. It would be better to perform the analysis of the whole mouse brain section if possible. Furthermore, I would strongly suggest that plots of the raw data are included in the manuscript. Ideally showing the whole slide and zoomed in signal dots with nuclei segmentation outlines. In addition, as average numbers of transcript per nucleus are reported it would be good to include the number of cells in each condition. If feasible, more genes should be included to get

© 2024. Published by The Company of Biologists under the terms of the Creative Commons Attribution License (https://creativecommons.org/licenses/by/4.0/).

a better estimate of the fold increase.

We agree with Reviewer 2, and this is something that was also pointed out by Reviewer 1. Please see point 1) of our response to reviewer 1 as well as the update figure.

2) The methods indicate that tissue sections with a thickness between 10 to 20 micrometers are used for this protocol. Are the cDNA-ISS and RNA-ISS conditions performed on the same thickness? Even though transcripts per nuclei are used in the calculation, section thickness could influence the counts, especially because the images are flattened with a maximum projection in Z. It would be helpful if the authors could report the number of nuclei in each section as proxy for the slice thickness.

We thank the reviewer for pointing this out. The experiments to compare RNA-ISS vs cDNA-ISS are performed on consecutive sections taken at the same thickness (10 micra). We clarified this in the main text.

3) Please include a protocol for the cDNA-ISS data used, or a literature reference if the dataset was already published. Also include a description of how the comparison is made in the methods.

We agree this part of the text needed clarification, and thank the reviewer for pointing this out. The protocol we used for cDNA-ISS is at: https://www.protocols.io/view/hybiss-hybridization-based-in-situ-sequencing-kqdg34357l25/v1 We now specified this in the materials and methods section. We generated fresh datasets using sections consecutive to the ones used for RNA-ISS, so to control for RNA degradation and tissue handling. We updated the text accordingly.

4) To further access sensitivity between cDNA-ISS and RNA-ISS an analysis similar to Lee et al. 2022 Scientific Reports, where the distribution of counts are directly compared (Fig 1b-e) would strengthen the manuscript. Furthermore, since many methods in this field assess their sensitivity by comparing to smFISH based data, this analysis would help place the new method in a common reference frame. Lastly, a comparison to 10X Xenium would also be interesting if possible.

We expanded on the analysis comparison, giving a better overview of the sensitivity increase in a revised Figure 2.
We agree with the reviewer that a comparison with smFISH is useful. From previous literature (summarized in https://www.annualreviews.org/docserver/fulltext/genom/24/1/annurev-genom-102722-092013.p df?expires=1713951927&id=id&accname=guest&checksum=16AC1301F35AC812E47B9530F9 0535ED), the capture efficiency of cDNA-ISS is estimated to be in the order of 1-5%. Assuming the results presented in the paper can be extrapolated fairly uniformly to the entire transcriptome, this would place the efficiency of open-source dRNA-ISS to about 2-10%.
According to a recent benchmarking, Xenium efficiency is likely similar to scRNAseq (30%), and the estimated capture efficiency of smFISH is above 90%. We introduced a brief discussion of these estimates in the text.

The introduction, Figure 1D and online code also covers cell segmentation and clustering of ISS data. However, no demonstrations of these steps are given in the manuscript. An addition of this, for instance on the chicken data, would demonstrate the full pipeline. Because these algorithms wrap existing tools that are independently published (PCIseq, Cellpose and Scanpy) a thorough evaluation is not needed in my opinion, but an example would complete the claims made in the introduction.

We now provide a small test dataset that can be used to try out the pipeline and its different modules. This includes all the steps (preprocessing, decoding, segmentation and single-cell clustering) as well as advanced features (deconvolution / denoising). We hope this satisfies the reviewer's request, increases the reproducibility of our work and softens the learning curve for new users.

Combining ISS with protein detection is a very promising capability which cannot easily be obtained with other spatially resolved transcriptomics techniques. However, in previous work the

© 2024. Published by The Company of Biologists under the terms of the Creative Commons Attribution License (https://creativecommons.org/licenses/by/4.0/).

Nilsson group already demonstrated that this was possible (Mbp protein in Lee et al. 2022 Scientific reports). Here this is again demonstrated with the GFAP protein. A stronger case would be made if multiple proteins are detected, because it is likely that not all epitopes are equally well preserved during the ISS protocol. However, this is a major effort and therefore toning down the claims would also suffice.

We agree with the reviewer that our claim was not strongly supported by the presented data. In the revised version we now include data from a larger number of protein stainings, collected by performing a CODEX multiplexed protein staining experiment after RNA-ISS. The full dataset is linked in the supplementary material.

The CODEX images can be explored either by using the scroller in the bottom left corner, or clicking on the 'layer' tab and selecting individual images for different antibody stainings.
https://lee2024supp.serve.scilifelab.se/mouse_ISS_CODEX.tmap

The authors suggest that next to EdU birth dating, other fluorescent reporters can be used in combination with ISS. However, if genetically encoded fluorescent proteins are used, will their fluorescence not interfere with the ISS signal? If they first need to be degraded would this affect the ability to use immunohistochemistry after barcode imaging?

We thank the reviewer for raising these very good points.
The answer to these questions depends on the nature of the specific reporter used. The imaging of the fluorescent reporters can be potentially performed at different stages in the experiment. In some cases (ie. GFP) the three strategies outlined below are valid.
A first option is to image the reporter just after fixation, before the library prep step. A second option is to image the reporter after RCA, but before the first cycle of fluorescent labeling.
In both cases the GFP fluorescence can be significantly reduced by denaturation with a 100% methanol treatment of 10 minutes at room temperature. This methanol-dependent denaturation of fluorescent protein is a well-documented phenomenon, and it is known to affect GFP derivatives as well as many fluorescent proteins in the red range (Tomato, mOrange, etc…). The level of residual background fluorescence might or might not affect the downstream steps, depending on the initial expression levels.
The third option is to methanol-denature the fluorescent proteins, proceed to RNA-ISS and posteriorly label the denatured fluorescent protein using immunohistochemistry. This antibody-based strategy is common practice in the Drosophila community when working with methanol-preserved early embryos expressing GFP, which typically lose up to 90% of the GFP fluorescence in the fixation step.

Clearly these strategies can be applied only to methanol-sensitive FPs whose fluorescence can be quenched without actually degrading the protein. If protein degradation is required to quench the reporter's fluorescence, we would suggest starting with library prep, pause the ISS protocol before the first detection cycle, image the desired proteins using immunohistochemistry, degrade the proteins using a proteinaseK treatment, and move on with the detection cycles.

Finally, a fourth option is to use a compatible set of ISS probes and genetically encoded fluorophore, so they are spectrally separated. Depending on the microscope's settings, this might imply having to sacrifice one of the channels used for decoding, perhaps reducing the multiplexing capability, to some extent.
We added these suggestions and thoughts to the supplementary manual, in the paragraph: *'Technical notes and considerations for successful ISS experiments'*.

The manuscript should better clarify to the reader that the basis for this protocol is HybISS (Gyllborg et al. NAR) rather than the original ISS protocol by Ke et al. 2013 Nature Methods.

Thanks for pointing this out. This is now corrected in the text.

The cross-reactive design of probes between species, is an interesting idea and can save resources in specific experimental situations where closely related species are studied.
However, it also suggests that RNA-ISS could suffer from more false positives. Could the authors elaborate on this and include a measurement of false positives?

© 2024. Published by The Company of Biologists under the terms of the Creative Commons Attribution License (https://creativecommons.org/licenses/by/4.0/).

We thank the reviewer for bringing this subject to our attention. Indeed, this is a matter we often discussed, but we don't have a clear take of what a good measurement of false positives would be. One option we often discussed, and seems to be the approach taken by some vendors in their reagent kits, is the inclusion of a negative control set of probes: probes that are not supposed to bind any target and should give zero signals. We show here a couple of examples of such "negative probes" against EGFP or Gal4, tested on ovaries of non-transgenic flies (hence lacking these reporter genes), showing a very low read count even before quality-filtering.

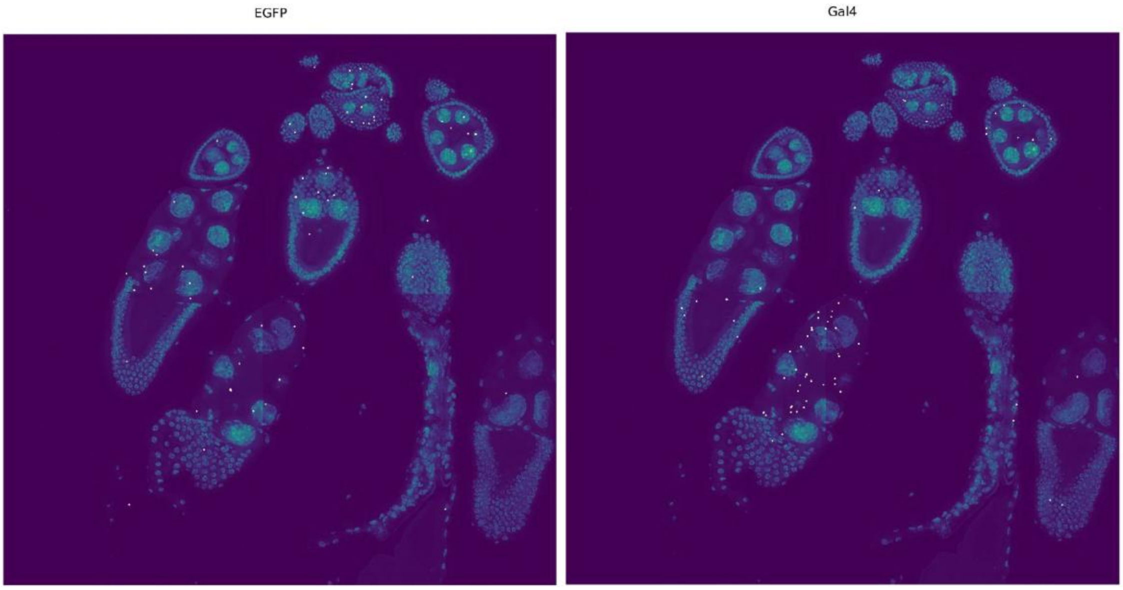

However, we struggle to see how the use of "negative probes" would intrinsically be more informative than validating the specificity of the assay against existing knowledge, hence our choice of not showing these data in the initial submission. The choice of performing our characterization on the fly ovary, where germ-line and somatic-line marker genes are well known and mutually exclusive, was driven by the need of a strong test case made of mutual internal controls, both positive and negative. We don't think in this context adding EGFP or GAL4 probes as negative control would add any further information. However, we acknowledge this could help in case the use of internal controls is not an option.

Another issue we often debated is what makes a good universal negative control. Or in other words: what's the source of potential experimental noise, and how do we tackle it rigorously in all of our experiments? We believe the sources of noise to be organism- and tissue-specific, and it's not always obvious to us whether a valid universal strategy to prevent noise exists.
Possible causes of noise might be, among others:

1. **expression of closely-related paralogues that spuriously bind the probes designed for another gene**. We try to address this to the best of our ability in the specificity-check step of probe design, excluding potentially cross-reactive targets within the transcriptome.
2. **Unexpected expression of pseudogenes or non-annotated genes with high sequence similarity to the chosen target genes**. This can be particularly problematic for poorly annotated transcriptomes, but we don't think a good universal solution exists, besides working with transcriptomes that are as complete as possible. Point 1 and 2 are also also discussed in response to Reviewer 1's comment about the root causes of the quantitative differences observed between mouse and rat.
3. **Template-independent ligation of padlock probes.** All ligases have some small degree of template-independent activity, and this is a potential source of noise. We try to prevent this by separating temporally hybridisation and ligation, and introduce a stringent washing step (neither of which was necessary for DNA-ISS), to remove most of the unhybridized or partially hybridized probes.The fact that cross-reactive design of probes is sometimes possible reinforces the notion that most of the specificity of our RNA-ISS assay is largely deriving from *hybridization specificity* and that the *ligation specificity* of

© 2024. Published by The Company of Biologists under the terms of the Creative Commons Attribution License (https://creativecommons.org/licenses/by/4.0/).

the padlock probes plays only a more secondary role, hence our choice to intervene aggressively in the washing step after hybridization. Perhaps negative probes might help address the efficacy of this third point, but we feel this would depend on the specific experiment, model and expected off-targets for the negative probes, which may vary depending on the used model system. We believe it's generally safer to have a couple of validated internal controls. When this is not possible, we agree that negative probes might be a valid approach, but we insist on the need of carefully designing these controls for each species, and keep in mind all the potential caveats.

We added these considerations in the Supplementary Manual, in the section '*Technical notes and considerations for successful ISS experiments*'.

For the simulation of the Macaque probes the authors note that a human probe panel has 74% matches, 12% has no-match and 14% has off-target matches to the Macaque transcriptome. However, a discussion and conclusion lack whether that would be enough to yield high quality data.

We thank the reviewer for bringing up this, which somehow relates to the puzzling results on the Rat vs Mouse comparison Reviewer 1 has pointed out.
The answer to this question largely depends on the scientist's need. Our suggestion would be to remove all the no-match and off-target probes from the experiment, and focus on the list of genes resulting from the single-matching probes. Is the resulting gene list satisfactory for the needs of the experimenter?
One hand, some genes will be excluded from the initial list, and it would be impossible for the scientist to probe them on Macaque, either because of no reactivity or cross-reactivity. Whether these missing genes are or not of crucial importance largely depends on the specific question the scientist will have and how important the analysis of those specific genes is.
On the other hand, a considerable number of genes in the panel will be recognized by a reduced number of probes. We acknowledge that this factor might potentially reduce the number of successful mRNA detections per cell, and produce weaker quantitative results. We estimate that, on average, the global detection efficiency in macaque would be 74% if the detection efficiency in human (assuming comparable gene expression levels and overall RNA integrity), so a notch closer to cDNA-ISS. However, as we've seen in the mouse Vs rat case, many variables might determine how one set of probes translates to a different animal. Depending on the specific question, this level of detection efficiency might or might not be sufficient, and our suggestion would be to do a test run.
We'd like to remark, however, that these human probes were not explicitly designed to be cross reactive on Macaques, and this expected efficiency drop could have been prevented by including cross-reactivity as a prior criterion in the probe design phase.

Figure 2 A-E, why is the background so different between the images?
Thanks for pointing this out. The background is different because in this specific experiment each gene was labeled with a unique fluorophore, and each channel has a specific signal/noise ratio in a given tissue. This is particularly visible in the '*tj*' panel, because these fly ovaries had a very bright autofluorescence in the AF488 channel (the fluorophore we used to detect that gene).

For none of the presented experiments the full dataset with all detected genes is shown simultaneously in a plot. Adding this would be valuable to show the alignment performance of the algorithm and give a better impression of the capabilities of the presented protocol and analysis code.

We think this is a very good suggestion. All the datasets can be now visualized at this permanent link: https://lee2024supp.serve.scilifelab.se/
When loading each of the datasets, by default all mRNA are simultaneously plotted to showcase the capability of the method.

Minor comments:
The keyword "Multi-omics" is a stretch because only one protein is detected using immunohistochemistry alongside the spatial transcriptome.

© 2024. Published by The Company of Biologists under the terms of the Creative Commons Attribution License (https://creativecommons.org/licenses/by/4.0/).

This was also pointed out by reviewer 1. We now added multiple protein stainings based on a RNAISS+CODEX dataset generated to support our “multi-omics” claim. The full ISS+CODEX dataset is visible at https://lee2024supp.serve.scilifelab.se/mouse_ISS_CODEX.tmap
We hope both reviewers agree that the new datasets support the claims.

Introduction:
Clarify that “sequencing-based” methods use Next Generation Sequencing, to prevent confusion with ISS.

This is now corrected in the text.

The authors describe the rolling circle amplification products to be “large”, “very bright” and “massively amplified”. It would be more informative to specify the (approximate) size, and the amount of amplification.

We agree with the reviewer, and we edited the text accordingly. We provide a size estimate for the amplicon (1 micron), and an amplification estimate (100-1000 copies).

On a similar note, please specify what size range a “large tissue section” is.
We agree with the reviewer, and we included in the text more specific information. We provide a realistic time budget based on our experience, 35 minutes for a 5 color scanning of tissue square of about 0.7 cm by side. We also tabulated imaging times of RNA-ISS against published osmFISH dataset (https://www.nature.com/articles/s41592-018-0175-z)

	**Comparison RNA-ISS to osmFISH**		
	Total # FOV	Total Area [mm^2]	Total time [min]
dRNA 20x	15372	52.70	26.04
osmFISH	9460	3.96	509.26

The authors list the detection of heavily fixed mRNAs as one of the limitations of ISS, and in the next paragraph summarize that the manuscript overcomes these limitations. However, no prove (or reference to previous work) is given that indicates that over fixing is an issue. Nor, is it demonstrated that the new protocol overcomes this specific issue.

This is a very good point. The decrease in ISS sensitivity caused by overly fixed samples is something that seems to be assumed knowledge, but we could not find references justifying the claim, except a few papers claiming RNA degradation as a consequence of excessive fixation (ie:https://www.nature.com/articles/srep21418, https://academic.oup.com/nargab/article/6/1/lqae008/7595396?login=true). Intriguingly, the Xenium test datasets released by 10x Genomics also show a reduced efficiency in FFPE samples Vs fresh frozen, but we would argue (in agreement with the reviewer) that this does not directly point to fixation as the problematic step. To our knowledge, the samples were not matched-controlled for other factors (ie, storage time).

We removed the claim from the text, and we believe this should be something to be explored more systematically in later work.

Results and discussion:

For the proof of principle experiments in Drosophila, mouse and chicken it would be helpful to see the images of the in situ reference databases side-by-side with the ISS data. A reference to the databases should also be included in the main text (Not only in the figure legend).

© 2024. Published by The Company of Biologists under the terms of the Creative Commons Attribution License (https://creativecommons.org/licenses/by/4.0/).

We agree with the reviewer. However, especially for *Drosophila*, the description of the expression patterns in the ovary predates the creation of modern databases. We could find pictures for *grk* and *slbo* in the Dresden Ovary Table (http://tomancak-srv1.mpi-cbg.de/DOT/main) but not for the other genes. We'd like to remark, however, that these genes were chosen precisely because they served as established markers for ovarian cells subpopulations during decades, and are well known to the fly community. If the reviewer agrees, at least for Drosophila, we'd prefer to sum up the information in a reference figure, and refer to the original publications rather than producing side-by-side images, because we might need permission from the publishers to include the original pictures in our paper.

vas https://www.sciencedirect.com/science/article/pii/0092867488902164?via%3Dihub
nos https://anatomypubs.onlinelibrary.wiley.com/doi/10.1002/aja.1001990204
tj https://www.nature.com/articles/ncb1058
grk https://www.sciencedirect.com/science/article/pii/S0092867405800935?via%3Dihub
slbo https://www.sciencedirect.com/science/article/pii/009286749290265E?via%3Dihub
en https://genesdev.cshlp.org/content/6/12b/2635

Not all the genes we probed are represented in the Allen Brain Atlas, so we opted to plot them side-by-side with the cDNA-ISS expression, and use that as a ground truth.
Finally, the data representation in the GEISHA database doesn't always cover the chosen genes for Chicken, particularly for the optic tectum.
We rephrased the statement in the relevant paragraph in the text to:
"For both species, the decoded expression patterns are localized, specific, and consistent with previous available knowledge whenever existing".

Include additional information on the homology between the engrailed gene in the different species. It would also be helpful to add percentages of nucleotides that differ inside the probed region between the species.

We added the following information to the supplementary data, explaining the difference in the target binding sites across species

Target 1:
Dmel TGGAGGATCGCTGCAGTCCACAGTCAGCGC
Dpse TtGAGGATCGCTGCAGTCCACAaaCAGCGC 3 mismatches Dvir
TtGAGGATCGCTGCAGTCCACAaTCAGCGC 2 mismatches

Target 2
Dmel GGCCAGTCCGCCACCCGCCTCCAATGCCTC
Dpse GGCCAGTCCaCCgCCCGCCTCgAATGCCTC 3 mismatches Dvir
GGCCAGTCCtCCgCCCGCCTCgAATGCaTC 4 mismatches

Target 3
Dmel CACAGAGGGCGGCAAGAACGAGATGTGGCC
Dpse CACcGAaGGCGGCAAGAACGAGATGTGGCC 2 mismatches Dvir
CACcGAcGGCGGCAAGAACGAGATGTGGCC 1 mismatch

Target 4
Dmel CTCCAGCGAGCAGTTGGCCCGCCTTAAGCG
Dpse CTCCAGCGAGCAaTTGGCCCGCCTcAAGCG 2 mismatches Dvir
CTCCAGCGAGCAaTTGGCACGCCTcAAGGT 2 mismatches

Target 5
Dmel GGAGGAGGAGCTCGAGATGCGCATGAACGG
Dpse GGAGGAGGAGCTCGAGATGCGCATGAACGG 0 mismatches Dvir
GGAGGAGGAGCTaGAGATGCGCATGAACGG 1 mismatch

© 2024. Published by The Company of Biologists under the terms of the Creative Commons Attribution License (https://creativecommons.org/licenses/by/4.0/).

Figure 3D-G, please add a label that this is rat brain. Possibly best to do this for all species in all figures.

This is now fixed in the figure.

Material and methods:

The sequences of all probes should be available in a supplemental file.
This was pointed out by Reviewer 1 as well. We now added this information.

Manufacturers all of chemicals and reagents should be included.
This information is now presented in the text and protocols.io

To which samples was TrueBlack applied?
Trueblack was applied to Chicken, the text is now updated
Which objective was used to image the RCPs?
20x for mouse and rat, 40x for Chicken and Drosophila. We clarified this in the text

How many Z-stack were imaged?

Imaging was performed using z-steps of 1um step size at 20x and 0.5 um at 40x. We clarified this in the text.

In the filtering of spots a quality score of 0.5 is used. How is this value determined and is there any ground truth data that is used to establish this value?

The formula for the calculation of the quality score is mentioned in the supplementary manual, but we agree with the reviewer that the rationale for its use should be made more explicit in the main text.

For each individual spot we extract the normalized intensity across all channels for each cycle. For every cycle, we divided the intensity value of the highest intensity channel ('true signal') by the sum of the intensities in all channels. Intuitively, values close to 1 represent a 'pure' signal, while lower values represent progressively uncertain identities. The minimum quality value for a 4-color decoding (as are all the experiments presented in the paper) is 0.25.
At the end of the decoding, each spot has *n* quality scores, where *n* is the number of detection cycles. In our filtering step (qmin >0.5) we keep the spots only if their quality was consistently alway above 0.5 in all the detection cycles.

From our experience, across a variety of datasets, experiments and imaging conditions, a quality minimum of 0.5 is a threshold that dramatically shifts the ratio between the *assigned reads* (spots whose identity is strictly and successfully matched against a decoding table) and the *non-assigned reads* (spots that fail to be assigned one of the allowed identities).
We typically plot the assigned Vs non-assigned read counts against some quality indicators (minimum and mean quality across cycle), to make an informed decision about our filtering criteria, and most often we find that a good balance is to filter on a Qmin=0.5. However, of course, this threshold can and should be tuned according to the specific experiment. A guide on how to explore the quality measures in relation to spots assignment is described in the Jupyter notebook in the ISS_decoding module, and the use of the relative functions is described in the Supplementary guide.

We added a brief explanation in the main text.

ISS manual:

In the filename "TileScan 0--Stage01--C03.tif" the index after "Stage" suggests that a maximum of 100 FOVs can be included (Stage00 to Stage99). Is the algorithm limited to 100FOVs?
We thank the reviewer for pointing this out. The algorithm is not limited to 100 FOVs. We regularly process samples with >200 FOVs. We clarified this in the manual.

© 2024. Published by The Company of Biologists under the terms of the Creative Commons Attribution License (https://creativecommons.org/licenses/by/4.0/).

Please give an indication of how large a large dataset is. This will help users prepare the hardware.

An experiment imaging a square of about 0.7 millimeters of side, imaged at 20x magnification using 5+1 colours (5 channels + DAPI) is about 600 GB. The bottleneck in our setup is data transfer, so we suggest a cloud/NAS infrastructure, to transfer the imaging data straight from the microscope onto the analysis workstation. However, in the past we often worked with external hard drives with some success. In this case we would advise using fast SSDs for all the processes from imaging to decoding.

We added these considerations in the Supplementary Manual, in the section '*Computer requirements*'.

Code:

The authors could consider putting a minimal raw dataset online for first time users to try.
We agree with the reviewer. We have now linked a testable dataset. Because of its relatively large size (50GB) the dataset is hosted at this link https://figshare.com/s/8e0c2bd43a3975fcff4a The link is currently private, but will be made public and searchable during the publication process in agreement with the publisher, pending acceptance of the manuscript.

We hope these changes satisfy all the reviewer's comments and requests.

On behalf of all the authors, we sincerely appreciated the positive tone of the reviews and the very constructive comments.

Sincerely, Marco Grillo

Second decision letter

MS ID#: DEVELOP/2023/202448

MS TITLE: Open-source, high-throughput targeted in-situ transcriptomics for developmental and tissue biology

AUTHORS: Hower Lee, Christoffer Mattsson Langseth, Sergio Marco Salas, Sanem Sariyar, Andreas Metousis, Eneritz Rueda Alana, Christina Bekiari, Emma Lundberg, Fernando Garcia Moreno, Marco Grillo, and Mats Nilsson

I have now received all the referees reports on the above manuscript, and have reached a decision. The referees' comments are appended below, or you can access them online: please go to BenchPress and click on the 'Manuscripts with Decisions' queue in the Author Area.

The overall evaluation is positive and we would like to publish a revised manuscript in Development, provided that the referees' comments can be satisfactorily addressed. Please attend to all of the reviewers' comments in your revised manuscript and detail them in your point-by-point response. If you do not agree with any of their criticisms or suggestions explain clearly why this is so. If it would be helpful, you are welcome to contact us to discuss your revision in greater detail. Please send us a point-by-point response indicating your plans for addressing the referees' comments, and we will look over this and provide further guidance.

Please address remaining reviewer concerns. I do not think additional demonstration of the pipeline is needed as suggested by reviewer 2, but you can include if possible. Please address the other concerns of reviewers 1 and 2 -while most can be addressed with additions to the text, there were questions raised regarding new data included in the revision. Please carefully address these

© 2024. Published by The Company of Biologists under the terms of the Creative Commons Attribution License (https://creativecommons.org/licenses/by/4.0/).

concerns with additions to the text and some additional quantification (to build confidence in the new data).

Reviewer 1

Advance summary and potential significance to field

I appreciate the author's effort in the revision of the article.
Overall, the authors have responded and corrected every point that was requested.
The new experiments showcased the power of RNA-ISS against cDNA-based approach and the flexibility of the method to combine it with immunofluorescence or EdU labelling. Below the authors will find few minor modifications.

Comments for the author

Minor modifications

- Please, add the references for the following claims
"These amplicons are large (approx. 1 micron) and chemically stable, contain concatemers of hundreds of copies of the original probe, …"

"To place these values in a common reference frame, smFISH is estimated to have a capture efficiency of over 90%, while droplet based scRNAseq efficiency rounds 30%, according to the 10x Chromium manual, …"
"… the last common ancestor of D. melanogaster and D. virilis is estimated to be 40 MYA, a time interval corresponding roughly to the split between old and new world monkeys."
"… using a set of padlock probes explicitly designed to work in both species (<20 MY apart), …"
"Among these, only the 30mers with a C or G in position 16 are kept: this is because T4RNAl2 has a slight positive efficiency bias towards terminal 3' G or C."

In "RNA-ISS recapitulates known mRNA expression patterns with high sensitivity"

- The text needs to be rearranged, so the order of the Figures matches the order in which they appear on the text (e.g. Fig 2D comes after Fig 2A).

- Check writing in "… illustrating that RNA-ISS is able to recapitulate the spatial expression of all genes with improved detection efficiency while maintaining specificity with RNA-ISS (Fig. 2C and 2D)."

In "RNA-based ISS is compatible with standard labeling techniques"

- Please, name the labeled proteins so readers can identify them on TissUUmaps
"… we ran RNA-ISS on a mouse coronal section using our new RNA-ISS chemistry on a previously described probe panel (Gyllborg et al., 2020; Ke et al., 2013; Lee et al., 2022) and posteriorly labeled a mouse coronal section with 11 barcoded antibodies, …"

Reviewer 2

Advance summary and potential significance to field

See first review.

Comments for the author

The authors have added most of the requested additional information and comparisons to previous/other methods to the manuscript. This places the advances in the proper context and aids

© 2024. Published by The Company of Biologists under the terms of the Creative Commons Attribution License (https://creativecommons.org/licenses/by/4.0/).

the reader to evaluate the work. The option to now interactively explore the data online is a nice addition to the manuscript.

Nevertheless, I still think the manuscript could benefit from a small demonstration of the full analysis pipeline, including cell segmentation and clustering of cells. To give readers a handle on the data quality and to demonstrate the claims made in the introduction. However, this is not an absolute necessity because it can be found on the Github tutorials to some extent, but it would improve the manuscript.

The addition of the new data and analysis raised two concerns that I would like the authors to address.

Point 1, RNA / cDNA comparison.
The 9 gene comparison between cDNA-ISS and RNA-ISS and the updated figure 2 is a very helpful addition to the manuscript and convincingly shows that RNA-ISS has higher counts on average. However, I think more proof is needed to show that these are not technical artifacts.

First, please add the standard deviation in the reported mean fold change between RNA-ISS and cDNA-ISS. Especially because the variation in fold change between genes is relatively high. What could the source of the variation be?

Second, in Figure 2C and Supplementary Figure 1E there are clearly more detected molecules outside the tissue area in the RNA-ISS condition compared to the cDNA-ISS condition. If these are false positives, this could bias, or in the worst-case dominate, the comparison substantially. Furthermore, also inside the tissue there seems to be more counts in areas where there are no, or few, counts in the cDNA-ISS method. For instance Rorb goes up in ventricle and hippocampus in the RNA-ISS, while this and other datasets do not suggest Rorb should be expressed there. Could the authors comment on the source of these dots? Is this ambient RNA that got stuck on the surface of the glass, or has it another technical source?

To prove the effect of the spurious signal on the fold change, could the authors please count the number of molecules outside the tissue for each of the 9 genes in RNA-ISS and cDNA-ISS, and correlate these values with the per-gene fold change to see if this explains the per-gene variability.

Next, could the authors calculate the count density outside the tissue and subtract this value of the count density inside the tissue, and then repeat the fold-change analysis. In case there is a homogeneous distribution of false-positives over the whole imaged area.

Furthermore, to exclude any other biases could the authors clarify if the exact same probes are used between the two conditions? The updated methods mention that the same number of probes is used, but not whether these are the exact same sequences.

Lastly, are all the settings and parameters used for the image analysis of the two conditions exactly the same?

Point 2, Protein co-detection

The combination of CODEX and ISS would be extremely powerful to study tissues and I commend the authors for trying to combine these methods.

However, while looking at the CODEX images of the 10 proteins (the manuscript mentions 11) on https://lee2024supp.serve.scilifelab.se/mouse_ISS_CODEX.tmap .
It seems like the antibody staining had some aspecific signal in the white matter of the brain. All antibodies, except GFAP and ACTB, label the white matter in the same way. Although, I am not familiar with all proteins and their localization, I would expect MOG to be located in the white matter but not others, like the microglia marker MRC1 and gray matter astrocytes marker S100B. Could the authors discuss the quality of the CODEX experiment and address this concern?

© 2024. Published by The Company of Biologists under the terms of the Creative Commons Attribution License (https://creativecommons.org/licenses/by/4.0/).

Furthermore, the GFAP staining presented in Figure 3M looks very similar to the other, potentially aspecific, protein stainings. While the GFAP staining in the CODEX experiment looks more like true GFAP labeling.
See for instance: https://doi.org/10.1007/s12031-020-01771-w and https://doi.org/10.1186/2040-2392-2-7 In these examples, note that there is no GFAP labeling in the white matter of the striatum, while this is visible in Figure 3M.

Furthermore, it would be nice to see the results of CODEX and ISS in a (supplementary) figure, and not just on the online data viewer.

Second revision

Author response to reviewers' comments

On behalf of all coauthors, I wish to thank both reviewers for the insightful comments and suggestions. We attach here a point-by-point response to their comments. We also edited the text for clarity and brevity, as requested by the editorial office.

Reviewer 1:

We thank Reviewer 1 for the positive comments and for pointing out the minor reviews addressed below:

Please, add the references for the following claims "These amplicons are large (approx. 1 micron) and chemically stable, contain concatemers of hundreds of copies of the original probe, …"

We added the following reference: Lizardi et al, 1998 (https://www.nature.com/articles/ng0798_225)

"To place these values in a common reference frame, smFISH is estimated to have a capture efficiency of over 90%, while droplet based scRNAseq efficiency rounds 30%, according to the 10x Chromium manual, …"

We edited the text, pointing to a single recent reference where the efficiency of the different technologies are systematically reviewed and compared (Salas, 2023) (https://www.biorxiv.org/content/10.1101/2023.02.13.528102v1)

"… the last common ancestor of D. melanogaster and D. virilis is estimated to be 40 MYA, a time interval corresponding roughly to the split between old and new world monkeys."

We added the relevant reference to the text:
https://academic.oup.com/mbe/article/13/1/132/1055488

"… using a set of padlock probes explicitly designed to work in both species (<20 MY apart), …"
We apologize for the oversight. The divergence time estimate for mouse and rat based on recent molecular data is actually larger (about 30 MY apart, https://academic.oup.com/mbe/article/18/5/777/1018665). We had initially followed an older estimate based on paleontological evidence (https://www.sciencedirect.com/science/article/pii/0047248480900627?via%3Dihub). We corrected the sentence in the text and added the pertinent reference.

"Among these, only the 30mers with a C or G in position 16 are kept: this is because T4RNAl2 has a slight positive efficiency bias towards terminal 3' G or C."

© 2024. Published by The Company of Biologists under the terms of the Creative Commons Attribution License (https://creativecommons.org/licenses/by/4.0/).

There's no reference in the literature pointing to this bias, the design criterion comes from our initial tests, not shown in the manuscript. However, this bias turned out to be very subtle, so (although this criterion is followed for the probe designed in the present work) we don't implement it anymore in the current lab's workflow, allowing us more flexibility in the probe placement, so to give transcripts a more even coverage across their length. We removed the claim about the efficiency bias from the text.

In "RNA-ISS recapitulates known mRNA expression patterns with high sensitivity"

- The text needs to be rearranged, so the order of the Figures matches the order in which they appear on the text (e.g. Fig 2D comes after Fig 2A).

Thanks for pointing this out. The text is now rearranged to match the figure order.

- Check writing in "… illustrating that RNA-ISS is able to recapitulate the spatial expression of all genes with improved detection efficiency while maintaining specificity with RNA-ISS (Fig. 2C and 2D)."

Thanks for pointing this out. We edited the text for clarity.

In "RNA-based ISS is compatible with standard labeling techniques"

- Please, name the labeled proteins so readers can identify them on TissUUmaps "… we ran RNA-ISS on a mouse coronal section using our new RNA-ISS chemistry on a previously described probe panel (Gyllborg et al., 2020; Ke et al., 2013; Lee et al., 2022) and posteriorly labeled a mouse coronal section with 11 barcoded antibodies, …"

We added a supplementary figure (also requested by Reviewer 2) with the CODEX antibody staining, and added the protein list to the text. We also apologize for a small oversight: for some reason the Synaptophysin staining was not included in the online viewer, but the online dataset is now updated with the missing staining.

Reviewer 2
We thank the reviewer for the evaluation of the new data and for pointing out their valid concerns. We acknowledge that a more detailed analysis might be beneficial to distinguish the real increase in efficiency of RNA-ISS from technical noise.

Here follows a point by point response to the comments:
The 9 gene comparison between cDNA-ISS and RNA-ISS and the updated figure 2 is a very helpful addition to the manuscript and convincingly shows that RNA-ISS has higher counts on average. However, I think more proof is needed to show that these are not technical artifacts. First, please add the standard deviation in the reported mean fold change between RNA-ISS and cDNA-ISS. Especially because the variation in fold change between genes is relatively high. What could the source of the variation be?

We thank the reviewer for pointing this out. We added the standard deviation in the reported mean fold change (average fold change =2.38 ; stdev=1.18).

As we mentioned in the previous round of response to reviewers, we believe a more systematic work on the probe design criteria might clarify the reasons behind the source of variation, and hopefully stabilize this variation in the upper range. Of all the factors that might play a role in determining a probe's efficiency, some are known to us and were included in our design pipeline (ie: optimal GC content, absence of homopolymeric stretches, etc…). Other factors are more complex and somewhat less obvious (secondary structure of target mRNAs in the cellular environment, partial pairing between different mRNA species, presence of RNA binding proteins, etc…). We speculate that cDNA-based methods might allow bypassing some of these constraints (ie: Reverse-transcriptases have strong strand displacement activity and might productively

© 2024. Published by The Company of Biologists under the terms of the Creative Commons Attribution License (https://creativecommons.org/licenses/by/4.0/).

resolve secondary structures or RNA pairing, allowing accessibility to RNA regions that would otherwise be tightly locked to probes in RNA-based approaches). We think more work is needed to characterize, understand and stabilize this variation towards the upper range.

Second, in Figure 2C and Supplementary Figure 1E there are clearly more detected molecules outside the tissue area in the RNA-ISS condition compared to the cDNA-ISS condition. If these are false positives, this could bias, or in the worst-case dominate, the comparison substantially.

We agree, this is visually striking and the reviewer is right in pointing this out. We don't believe these extra-tissue dots are false positives. Our argument for this is based on 2 elements: first, the level of detection of negative control padlock probes (as shown in the supplementary manual for the GAL4 negative probes) is very low. However, we acknowledge that it might be difficult to generalize results from negative probes, as we discussed in the previous round of revisions.

Our second element against these signals arising from unspecific ligation of padlock probes is that the abundance of mRNA dots outside the tissue is strongly correlated with the detected gene expression levels inside it (ie: high-expressors show high number of extra-tissue dots). One might of course argue, as the reviewer hints, that the high detection efficiency inside the tissue could be an artifact of the false positive ligations happening outside, and the increased sensitivity is a consequence of poor specificity.

Our best argument to solve this potentially circular argument is the following: the amount of extra-tissue dots in RNA-ISS is strongly correlated with the expression levels detected inside the tissue when using cDNA-ISS (see below for a more detailed analysis). To us, this indicates a directional causal link between the gene expression levels and the abundance of extra-tissue spots and suggests that the extra-tissue signal is real mRNA, most likely coming from diffusion/smearing of (partially degraded?) RNAs during the cutting and handling of the sections. In our experience, a similar effect is also often visible when using the CARTANA HS-ISS kit, especially on high expressors, and we believe it's a feature of RT-free methods, which are probably less impaired by mRNA fragmentation than cDNA-based approaches.

Furthermore, also inside the tissue there seems to be more counts in areas where there are no, or few, counts in the cDNA-ISS method. For instance Rorb goes up in ventricle and hippocampus in the RNA-ISS, while this and other datasets do not suggest Rorb should be expressed there.

We understand the reviewer's concern. We warn, however, against using cDNA-ISS as ground truth: because of its lower capture efficiency, lack of detection might sometimes be confused with lack of expression. The same applies to traditional chromogenic in-situ hybridisation, where a low-level staining is sometimes difficult to interpret in terms of absolute number of molecules Vs background noise.

As the reviewer suggests, these Rorb dots outside of the expected locations might be indeed diffusing mRNAs from nearby locations. However, we'd like also to point out that that Rorb's mRNA seems to be expressed (although at relatively low levels) in the hippocampus, according to these scRNAseq and MERFISH datasets:

scRNAseq:
https://rb.gy/f118vj

MERFISH:
https://rb.gy/x7nw3s

Could the authors comment on the source of these dots? Is this ambient RNA that got stuck on the surface of the glass, or has it another technical source?

Ambient RNA that gets stuck on the surface of the glass is certainly problematic, as we have discussed above when addressing the presence of dots outside the tissue. However, with the current data we can't surely tell whether that's the causative factor for Rorb detection, at least for the case of the hippocampus.

© 2024. Published by The Company of Biologists under the terms of the Creative Commons Attribution License (https://creativecommons.org/licenses/by/4.0/).

To prove the effect of the spurious signal on the fold change, could the authors please count the number of molecules outside the tissue for each of the 9 genes in RNA-ISS and cDNA-ISS, and correlate these values with the per-gene fold change to see if this explains the per-gene variability.

To perform the suggested analysis we used TissUUmaps to manually draw a ROI around the tissue using the DAPI signal as a reference to outline the tissue area. This is what we call "inside the tissue" in the following paragraphs. We then designed a second ROI spanning the actual imaged area, avoiding the black regions added by the stitching process. Creating this second ROI is crucial to make sure the analysis is not biased by an uneven representation of black areas in the images across conditions. The area contained between the external and internal ROI is called "outside the tissue" in the following paragraphs. We then used these ROI masks to assign a categorical variable to the reads, representing whether each read is contained in the "inside" or in the "outside" the tissue areas. We finally performed a correlation analysis on a gene-by-gene level.

The results are as follows:

1. The number of "outside" reads of RNA-ISS per gene and the corresponding raw fold change (dRNA/cDNA all slide) are not correlated (Pearson Correlation: -0.39, P-value: 0.29).
2. The ratio between RNA/cDNA "outside" counts does not correlate with the fold change (Pearson Correlation: -0.02, P-value: 0.95).
3. The ratio between RNA/cDNA "inside" counts correlates with the fold change (Pearson Correlation: 0.99, P-value: 5*10^-15)

We believe these results suggest that most of the total fold-change is explained by reads on the tissue, and that the reads outside the tissue contribute only marginally to the fold change increase.

Finally, we noticed how the "outside counts" for a given gene seemed to be correlated with its expression inside the tissue (high expressors tend to show more extra-tissue dots). To rule out the circular argument exposed in the previous paragraph, we assessed the correlation between "RNA-ISS outside counts" Vs "cDNA-ISS inside counts". This should allow us to inspect a causal link between the levels of gene expression and its extra-tissue detection abundance.
These 2 measures are indeed correlated (Pearson Correlation: 0.88, P-value: 0.001).

To us this suggests that the "outside reads" in RNA-ISS are proportional to the real gene expression levels and likely arising from real mRNA detection events. We speculate, as hinted above, that these signals are detected because RNA-ISS might be more capable than cDNA-ISS of detecting partially degraded mRNAs sticking to the glass.

Next, could the authors calculate the count density outside the tissue and subtract this value of the count density inside the tissue, and then repeat the fold-change analysis. In case there is a homogeneous distribution of false-positives over the whole imaged area.

As outlined before, we created the 2 ROIs to cover the tissue and the extra-tissue space. We then used the ROI contours to calculate the total area of each ROI across conditions and, for each gene, computed the read density by area unit, inside and outside the tissue.

As suggested by the reviewer, we then subtracted the "outside density" from the "inside density", both for cDNA and dRNA. We then analyzed the ratio between the dRNA "corrected density" Vs its cDNA counterpart. This correction reduced the fold change increase of RNA-ISS vs cDNA-ISS to about 1.96 from an initial value of 2.38. Also, it reduced the standard deviation to 1.04 from an initial 1.18.

Furthermore, to exclude any other biases could the authors clarify if the exact same probes are used between the two conditions? The updated methods mention that the same number of probes is used, but not whether these are the exact same sequences.

© 2024. Published by The Company of Biologists under the terms of the Creative Commons Attribution License (https://creativecommons.org/licenses/by/4.0/).

cDNA-ISS and RNA-ISS probes have different sequence requirements, so the chosen target sequences are different by design. We picked at random, among all the allowed targets on mRNA or cDNA, the same number of probes for both techniques, without any particular matching criterion. We acknowledge this might explain at least part of the observed variation, and perhaps a systematic analysis would reveal that the most efficient RNA-ISS probes have some distinguishing feature that we haven't accounted for and could be included in a later design optimization.

We added the following paragraph to summarize the analysis described above:

A careful analysis of the detected signal spots across methods revealed that RNA-ISS captured a higher number of spots outside the tissue compared to cDNA-ISS (FIG supp?), prompting the need to rule out technical artifacts. the higher sensitivity of RNA-ISS does not seem to be explained by this increased detection outside the tissue (Pearson Correlation: -0.39, P-value: 0.29), but does instead correlate with the RNA/cDNA ratios inside it (Pearson Correlation: 0.99, P-value: 5*10^-15). This suggests that reads outside of the tissue have a minor impact on the fold change increase. Furthermore, RNA-ISS counts outside the tissue are correlated with the expression levels inside it (as detected by cDNA-ISS) (Pearson Correlation: 0.88, P-value: 0.001), suggesting that these spots might be mRNA molecules smeared over the glass during the sample handling, and are not artifacts generated by a spurious activity of the ligase.

Lastly, are all the settings and parameters used for the image analysis of the two conditions exactly the same?

Yes, the images were acquired in exactly the same way, on the same day, same microscope with identical imaging settings (most notably, exposure time and LED power), on consecutive tissue sections. The analysis was performed exactly in the same way, with unchanged spot detection settings across methods. This is now clarified in the text, under "cDNA/RNA ISS comparison", by the addition of the following text: The images were acquired on consecutive sections, using the same microscope, unchanged imaging settings, and analyzed using the same image analysis pipeline with identical detection criteria.

Part 2 codex experiments:

We thank the reviewer for their appreciation of our attempt at integrating CODEX and ISS on the same samples. We acknowledge the issue with the background autofluorescence, and we wish to clarify our interpretation of the data: this issue seems more of a CODEX-specific problem than an artifact due to performing CODEX after ISS.

All antibodies were initially tested in a regular immunofluorescence experiment (ie. using secondary antibodies for detection). After this first validation, the antibodies were then conjugated with DNA barcodes and tested again, this time using the DNA barcodes for staining. With this multiple step selection process, we aimed to ensure the selection of high quality and specific protein targets.
However, in a few cases, as the reviewer observed, we could see background autofluorescence in the white matter after conjugation (specifically in the corpus callosum region). We chose to incorporate these markers to the panel because they were highly specific in other regions of the brain. Examples for these are CD31, LMNB1 and Ki67. The CD31 staining apart from the white matter autofluorescence was highly specific with visible vessel structures overlapping with descriptions in the literature (1). On the same line, the LMNB1 antibody stained the nuclear lamina specifically (visible around the nucleus). Ki67 antibody was also specific with the nuclear staining pattern in the lateral ventricular area matching previous descriptions in the literature (2) (attached Figure 1). Apart from these three markers, in the MRC1 staining, autofluorescence in the white matter is most likely an artifact of over-exposure, as MRC1 is not an abundant protein in the healthy mouse brain. Also, MRC1 is used as a marker for border-associated macrophages (BAMs) rather than microglia. In our stainings, we see BAMs in the outer layer of the brain, which is in concordance with the literature (3) (attached Figure 2).

All the other markers (CNP, NEFL, S100B) apart from the ones mentioned by the reviewer as specific stainings (ACTB, GFAP, MOG) are also highly specific. Similar to MOG, CNP is a marker associated with myelin sheaths. As the corpus callosum is rich in myelinated axons, the observed

© 2024. Published by The Company of Biologists under the terms of the Creative Commons Attribution License (https://creativecommons.org/licenses/by/4.0/).

CNP staining in the corpus callosum region is as expected which is overlapping with the literature (https://www.proteinatlas.org/ENSG00000173786-CNP/brain). NEFL is a marker for neurofilaments which are important components of the neuronal cytoskeleton. The corpus callosum contains numerous axons that are rich in neurofilaments which also explains the staining we observe in the corpus callosum region. The NEFL staining is also overlapping well with the literature available (https://www.proteinatlas.org/ENSG00000277586-NEFL/brain). In addition, we observed a minor autofluorescence in corpus callosum region in the S100B staining and the patterns we observed in the other regions of the brain was overlapping with the literature (https://www.proteinatlas.org/ENSG00000160307-S100B/brain).

We are still working on improving our stainings and incorporating applications to remove the autofluorescence arising from antibody conjugation of DNA barcodes. We hope this integration experiment can be considered as a step towards multi-modal integration approaches.

To clarify, we added the following text to the Materials and Methods section, under "Antibody conjugation": In a few cases we could detect some background autofluorescence in the white matter (specifically in the corpus callosum), arising after DNA conjugation to the antibodies. In these cases we chose to use the antibodies whenever they retained highly specific labeling in other regions of the brain. Examples for these are CD31, LMNB1 and Ki67.

We then added the following text in the results, when presenting the CODEX+ISS data:
For some of the antibodies we could detect, together with the specific labeling, some unspecific staining in the corpus callosum (Supplementary Figure 3). This unspecific staining seems to arise as a consequence of the DNA-conjugation to the CODEX antibodies, and it's not specifically produced by the combined CODEX+ISS workflow (see materials and methods, "Antibody conjugation" paragraph)

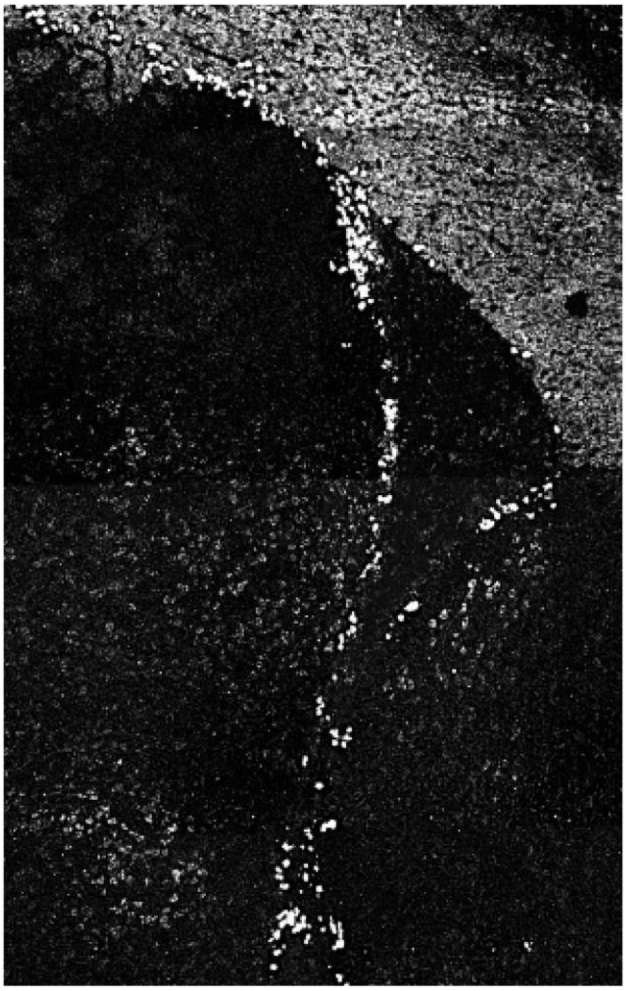

© 2024. Published by The Company of Biologists under the terms of the Creative Commons Attribution License (https://creativecommons.org/licenses/by/4.0/).

Figure 1-Higher Ki67 staining in the lateral ventricular area.

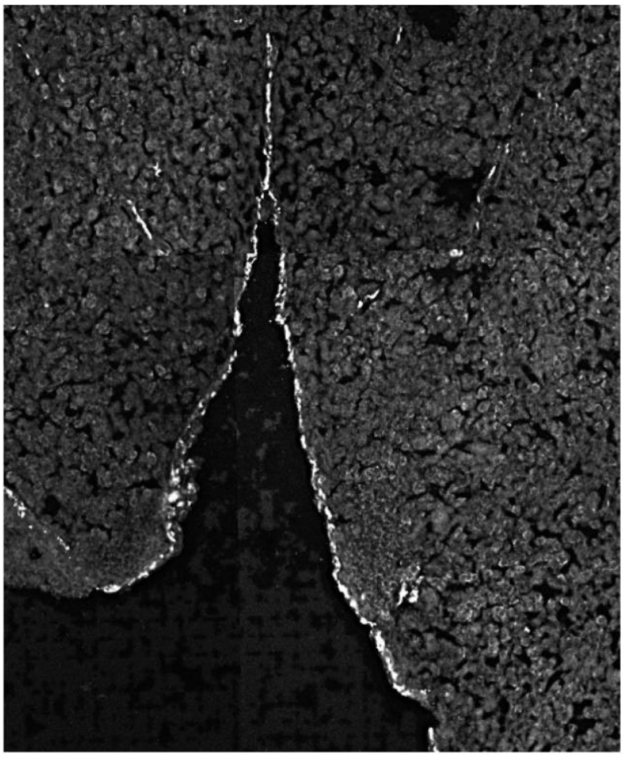

Figure 2-BAMs in the outer region of the brain tissue.

Regarding the GFAP staining in Figure 3M, the reviewer is right in pointing out those concerns. Something seems off with that particular staining, and we apologize for not spotting the problem earlier. We went back to the raw data and checked a replicate of the same experiment on a consecutive section, which shows a more convincing staining. We have now edited figure 3M to show this replicate instead.

Finally, we included a demonstration of data clustering and visualization in the Optic Tectum TissUUmaps data viewer. Because the cell density in the optic tectum is very high, instead of Cellpose-based segmentation we chose to bin the reads into hexagonal regions of size 100 micrometers. In our experience, Cellpose doesn't perform optimally on such cell-dense images, and leads to segmentation artifacts, unless specifically re-trained.
We then proceeded to analyze and cluster these bins. When loading the Optic Tectum datasets, now by default the clustered bins are also plotted on the image, and the user can opt to see them and switch between this view and the raw reads. Through the use of the DGE plugin, the user can visualize the differentially expressed genes for each cluster interactively. The UMAP can be visualized interactively using the FeatureSpace plugin.

We hope our response satisfies all the reviewers' requests.

1) Chen MB, Yang AC, Yousef H, Lee D, Chen W, Schaum N, Lehallier B, Quake SR, Wyss-Coray T. Brain Endothelial Cells Are Exquisite Sensors of Age-Related Circulatory Cues. Cell Rep. 2020 Mar 31;30(13):4418-4432.e4. doi: 10.1016/j.celrep.2020.03.012. PMID: 32234477; PMCID: PMC7292569.
2) Prabhakar S, Cheah PS, Zhang X, Zinter M, Gianatasio M, Hudry E, Bronson RT, Kwiatkowski DJ, Stemmer-Rachamimov A, Maguire CA, Sena-Esteves M, Tannous BA, Breakefield XO. Long-Term Therapeutic Efficacy of Intravenous AAV-Mediated Hamartin Replacement in Mouse Model of Tuberous Sclerosis Type 1. Mol Ther Methods Clin Dev. 2019 Aug 16;15:18-26. doi: 10.1016/j.omtm.2019.08.003. PMID: 31534984; PMCID: PMC6745533.
3) Silvin A, Qian J, Ginhoux F. Brain macrophage development, diversity and dysregulation in health and disease. Cell Mol Immunol. 2023 Nov;20(11):1277-1289. doi: 10.1038/s41423-023-01053-6. Epub 2023 Jun 26. PMID: 37365324; PMCID: PMC10616292.

© 2024. Published by The Company of Biologists under the terms of the Creative Commons Attribution License (https://creativecommons.org/licenses/by/4.0/).

Third decision letter

MS ID#: DEVELOP/2023/202448

MS TITLE: Open-source, high-throughput targeted in-situ transcriptomics for developmental and tissue biology

AUTHORS: Hower Lee, Christoffer Mattsson Langseth, Sergio Marco Salas, Sanem Sariyar, Andreas Metousis, Eneritz Rueda Alana, Christina Bekiari, Emma Lundberg, Fernando Garcia Moreno, Marco Grillo, and Mats Nilsson

I am happy to tell you that your manuscript has been accepted for publication in Development, pending our standard publication integrity checks.

Reviewer 2

Advance summary and potential significance to field

-

Comments for the author

The authors convinced me with their argumentation and extra analysis, that the increase in counts is not due to technical artifacts, and the RNA-ISS is more sensitive than cDNA-ISS.

I agree with the authors that the issues with the antibody staining are CODEX related, rather than ISS-CODEX. Applying CODEX is evidently not trivial, and I appreciate that the authors now make this clear in the manuscript, so that readers can properly evaluate the challenge if they would try this.

All in all, I would like to recommend the manuscript for publication. I would also like to commend the authors again on their efforts to make the method open-source.

© 2024. Published by The Company of Biologists under the terms of the Creative Commons Attribution License (https://creativecommons.org/licenses/by/4.0/).
